# Crosstalk Between NK Cell Receptors and Tumor Membrane Hsp70‐Derived Peptide: A Combined Computational and Experimental Study

**DOI:** 10.1002/advs.202305998

**Published:** 2024-01-31

**Authors:** Mina Yazdi, Morteza Hasanzadeh Kafshgari, Fatemeh Khademi Moghadam, Vahid Zarezade, Rupert Oellinger, Mohammad Khosravi, Stefan Haas, Cosima C. Hoch, Alan Graham Pockley, Ernst Wagner, Barbara Wollenberg, Gabriele Multhoff, Ali Bashiri Dezfouli

**Affiliations:** ^1^ Pharmaceutical Biotechnology Department of Pharmacy Ludwig‐Maximilians‐Universität (LMU) 81377 Munich Germany; ^2^ Heinz‐Nixdorf‐Chair of Biomedical Electronics Campus Klinikum München rechts der Isar TranslaTUM Technische Universität München 81675 Munich Germany; ^3^ Department of Biology Faculty of Science Shahid Chamran University of Ahvaz Ahvaz 6135783151 Iran; ^4^ Behbahan Faculty of Medical Sciences Behbahan 6361796819 Iran; ^5^ Institute of Molecular Oncology and Functional Genomics School of Medicine Technische Universität München 81675 Munich Germany; ^6^ Central Institute for Translational Cancer Research (TranslaTUM) School of Medicine Technische Universität München 81675 Munich Germany; ^7^ Department of Pathobiology Faculty of Veterinary Medicine Shahid Chamran University of Ahvaz Ahvaz 6135783151 Iran; ^8^ Department of Radiation Oncology School of Medicine Technische Universität München 81675 Munich Germany; ^9^ Department of Otorhinolaryngology School of Medicine Technische Universität München 81675 Munich Germany; ^10^ John van Geest Cancer Research Centre School of Science and Technology Nottingham Trent University Nottingham NG11 8NS UK

**Keywords:** Adoptive NK cell‐based immunotherapy, CRISPR/Cas9, Computational analysis, Heat shock protein 70, NK cell receptors

## Abstract

Natural killer (NK) cells are central components of the innate immunity system against cancers. Since tumor cells have evolved a series of mechanisms to escape from NK cells, developing methods for increasing the NK cell antitumor activity is of utmost importance. It is previously shown that an ex vivo stimulation of patient‐derived NK cells with interleukin (IL)‐2 and Hsp70‐derived peptide TKD (TKDNNLLGRFELSG, aa450‐461) results in a significant upregulation of activating receptors including CD94 and CD69 which triggers exhausted NK cells to target and kill malignant solid tumors expressing membrane Hsp70 (mHsp70). Considering that TKD binding to an activating receptor is the initial step in the cytolytic signaling cascade of NK cells, herein this interaction is studied by molecular docking and molecular dynamics simulation computational modeling. The in silico results showed a crucial role of the heterodimeric receptor CD94/NKG2A and CD94/NKG2C in the TKD interaction with NK cells. Antibody blocking and CRISPR/Cas9–mediated knockout studies verified the key function of CD94 in the TKD stimulation and activation of NK cells which is characterized by an increased cytotoxic capacity against mHsp70 positive tumor cells via enhanced production and release of lytic granules and pro‐inflammatory cytokines.

## Graphical abstract

This work investigates the interaction between the functional part of Hsp70 protein (TKD) and NK cell receptors through in silico and in vitro studies. CD94 activating receptor plays a major role in the TKD‐mediated NK cell stimulation. The CD94 complex with TKD part of membrane Hsp70 overexpressed on tumor cells is a key step to trigger the cytolytic signaling cascade of NK cells leading to enhanced release of lytic granules.

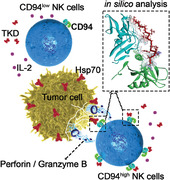

## 1 Introduction

Considering the importance of the immune system in the recognition and control of cancer, immunotherapy has gained tremendous attention in recent years.^[^ ^1^ ^]^ However, the clinical efficacy of immunotherapies is tempered by multiple factors such as tumor heterogeneity and the immunosuppressive nature of the tumor microenvironment (TME) which foster a broad variability in patient responses and treatment outcomes.^[^ ^2^ ^]^ The success of cancer immunotherapy is highly dependent on the presence and expression of tumor‐relevant and tumor‐specific targets. Hence, the emphasis has been on developing strategies to boost the selective natural killer (NK) and T cell targeting of tumors by better understanding and improving receptor‐ligand interactions.^[^ ^3^ ^]^ Noteworthy, novel approaches for selective tumor targeting are encouraged in all fields related to cancer therapy.^[^ ^4^ ^]^

NK cells, morphologically characterized as large granular lymphocytes, are important drivers of innate immune responses due to their capacity to recognize and kill pathogen‐infected and tumorigenic cells without the need for prior antigen stimulation.^[^ ^5^ ^]^ NK cells also play an indispensable role in bridging innate and adaptive immunities by exhibiting a number of immunoregulatory functions such as cytokine secretion.^[^ ^5^ ^]^ CD3^−^NK cells broadly comprise two major subsets differing in the cell‐surface density of CD56 and/or CD16 expression. The cytotoxic activity of NK cells is mainly mediated by the CD56^low^CD16^+^ NK cell subpopulation, whereas cytokine production is attributed to the CD56^high^CD16^−^ cell subpopulation which is predominantly found in the peripheral blood and in inflamed tissues and lymph nodes, respectively.^[^ ^6^ ^]^ In the interaction with tumor cells, NK cell functions are regulated by a delicate interplay between activating and inhibitory signals transduced by multiple germline‐encoded surface receptors which are activated after contact with relevant tumor antigens/ligands.^[^ ^7^ ^]^ Although the formation of the immunological synapse which allows NK cell receptors to bind their specific ligands is a transient phenomenon, it is a critical step in NK cell activation and the selective killing process. The transitory status of the immunological synapse can lead to “serial killing” which involves a fraction of appropriately activated NK cells becoming detached from their targets to enable their attack on other relevant target cells.^[^ ^8^ ^]^ NK cell activation and target cell engagement induce direct cancer cell death by different mechanisms including cytotoxic granule and death receptor‐mediated cell lysis as well as enabling complement‐ and/or antibody‐dependent cell‐mediated cytotoxicity (ADCC).^[^ ^9^ ^]^ Moreover, activated NK cells can produce an array of immunoregulatory cytokines and chemokines to recruit immune cells into the tumor microenvironment and thereby further amplify and orchestrate the immune response.^[^ ^10^ ^]^ Accompanied by receptor‐priming signals, the initial anti‐tumor response elicited by NK cells is supported by adaptive immune cells through contact and paracrine/exocrine signaling.^[^ ^10^ ^]^ For instance, in vaccine‐induced immunity, the quantity and quality of NK cells can be promoted by interleukin (IL)−2 secreting memory T cells.^[^ ^11^ ^]^ Additionally, it has been shown in a mouse model that the delivery of IL‐15 to NK cells by dendritic cells is vital for NK cell priming and their full activation which involves interferon‐gamma (IFN‐γ) production and cytolysis.^[^ ^12^ ^]^ Cytokine‐activation of autologous and allogeneic NK cells (ex vivo or via systemic administration) has therefore become a focus for the development of effective cell‐based adoptive immunotherapy.^[^ ^13^ ^]^ In addition to cytokine and contact‐dependent activation, immunogenic peptides can also regulate NK cell activity via receptor‐specific binding.^[^ ^14^ ^]^ Regardless of the many attempts to standardize the approaches for ex vivo expansion/stimulation of NK cells for adoptive immunotherapy, major challenges for successful translation into clinical practice remain.

Developments in the fast‐moving field of targeted immunotherapy benefit from the identification of relevant tumor‐associated antigen (TAA) and tumor‐specific antigen (TSA) targets. Whereas TSAs are selectively found on the cell surface of cancer cells, both normal cells and tumors express TAAs, albeit expression is higher on malignantly transformed cells.^[^ ^3^ ^]^ The specificity, anatomical location, quantitation, and functional affinity of TAAs can support the efficiency and safety of antigen‐based targeted therapies.

The stress‐inducible heat shock protein 70 (Hsp70) is a cytosolic protein of almost all nucleated cells but is predominantly overexpressed in the majority of solid and hematological malignancies.^[^ ^15^ ^]^ In malignant cells, the tumor‐specific glycosphingolipid globotriaosylceramide, Gb3, enables the localization of Hsp70 on the plasma membrane of cancerous cells, as a consequence of which mHsp70 can be considered as a TSA.^[^ ^15^, ^16^ ^]^ The cell surface density of membrane Hsp70 (mHsp70) expression positively correlates with advanced tumor stages, metastasis, and poor prognosis.^[^ ^17^ ^]^ Hsp70 can also be actively released from tumors in the context of extracellular vesicles (EVs), levels of which can reflect the mHsp70 expression status of their parental cells and tumors.^[^ ^18^ ^]^ Importantly, tumors expressing mHsp70 can be targeted and effectively eradicated by cytotoxic NK cells that have been triggered to recognize mHsp70 by incubation with a 14‐mer Hsp70‐derived peptide (TKDNNLLGRFELSG, “TKD”) in combination with low‐dose IL‐2^[^ ^19^ ^]^ or genetically engineered to recognize mHsp70.^[^ ^4a^ ^]^ A monoclonal antibody (mAb) cmHsp70.1 which was produced in our laboratory is able to recognize mHsp70 on the cell surface of viable tumor cells with an intact plasma membrane.^[^ ^20^ ^]^ The 8‐mer epitope of this mAb (NLLGRFEL) identified by pepscan analysis is part of the extended Hsp70‐derived peptide TKD (TKDNNLLGRFELSG) which is presented on the outer cell surface of tumor cells.^[^ ^20^ ^]^ Regarding these findings, we assume that the TKD sequence which contains the cmHsp70.1 antibody epitope is presented on the cell surface of tumor cells and might also be recognized by TKD/IL‐2‐activated NK cells. The synergistic effect of such a cytokine‐peptide cocktail to drive NK cell function can ensure the safety and efficiency of the treatment modality, which has the highest priority in clinical application. The mHsp70 is therefore an excellent, selective, actionable tumor‐specific target for appropriately targeted cell‐based immunotherapies.

As ligand‐receptor interactions are key prerequisites for functional communication between tumors and NK cells, reducing clinical off‐target toxicities requires highly selective protein/peptide drugs and a comprehensive understanding of ligand/receptor complexes and downstream molecular mechanisms of related biological pathways. In this study, in silico analyses such as molecular docking and molecular dynamics simulation were used to characterize the interaction of TKD, as the functional and interaction site of Hsp70, compared to the whole Hsp70 protein to different NK cell receptors. The in silico findings were then covalidated and confirmed in vitro using primary NK cells isolated from the peripheral blood mononuclear cells (PBMCs) of healthy donors. A strong correlation between computational and experimental findings indicated that the C‐type lectin receptor CD94 is the most likely active site for the TKD binding to NK cells. The involvement of CD94 in Hsp70/TKD interaction and the subsequent stimulation and effector function was confirmed by CD94 antibody blocking studies and in wild‐type and CRISPR/Cas9‐CD94 knockout NK cells.

## 2 Results and Discussion

### 2.1 Molecular Docking Analysis of Hsp70‐Derived TKD Peptide Interactions with Receptors on Primary NK Cells

**Figure 1:**
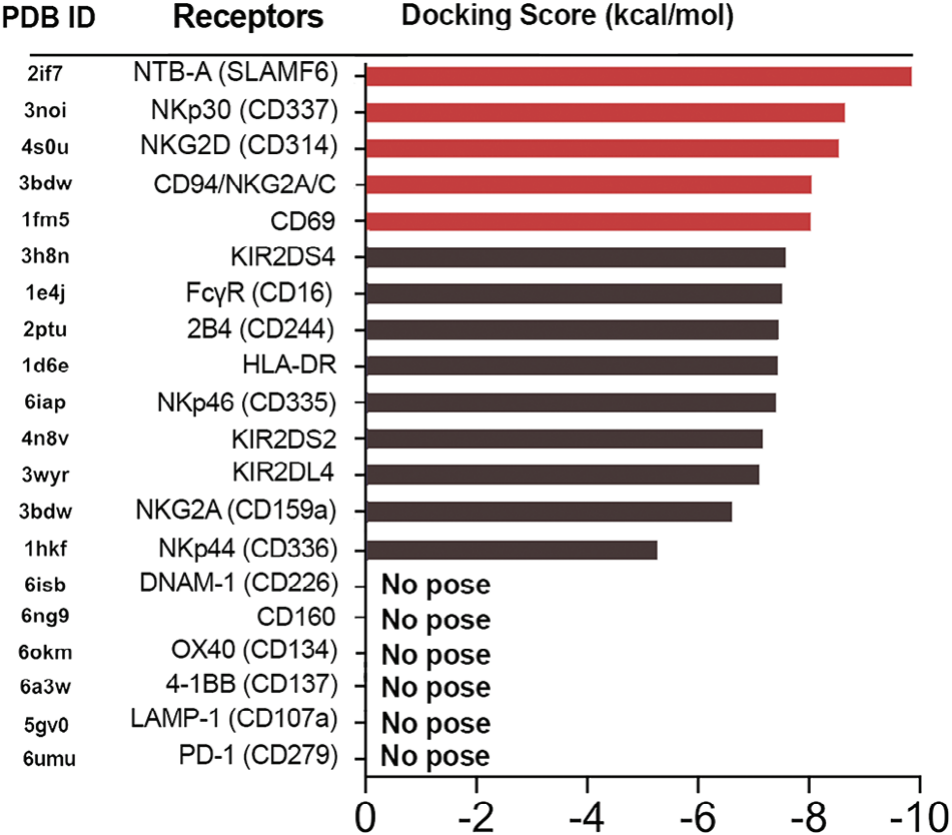
Molecular docking score (kcal/mol) of TKD against NK cell receptors using Schrödinger Software. PDB ID, protein data bank identity.

Computational approaches in drug design and discovery provide quantitative predictions of molecular interactions^[^ ^21^ ^]^ and herein molecular docking was used to interrogate intramolecular events (e.g., binding energy, affinity of the ligand at the active site of the target) upon binding of TKD peptide with the well‐characterized NK cell receptors. It is worth noting that ≈40% of protein‐protein interactions are mediated by short peptide sequences that mimic the functionality of the source protein and can be ideal therapeutic candidates.^[^ ^22^ ^]^ The TKD peptide was docked to the ligand binding site of the NK cell receptors using the Glide dock of the Schrödinger Maestro, and the receptors were then ranked according to the energy scoring function (**Figure** **1**). The best binding modes were selected based on the molecular docking score; the higher the negative energy value, the more favorable the binding mode. Based on this approach and considering a binding energy score of ≤−8 kcal mol^−1^, the activating receptors NTB‐A, NKp30, NKG2D, CD94/NKG2A/C, and CD69 with a binding energy score of −8 kcal mol^−1^ and above exhibited a comparatively better binding affinity than all other receptors. The optimal binding affinity of TKD towards these receptor candidates was re‐confirmed using the Hierarchical Flexible Peptide Docking (HPEPDOCK) server (Table S1, Supporting Information). As shown previously,^[^ ^23^ ^]^ TKD peptide has identical effects as the full‐length Hsp70 protein at equimolar ratios on NK cells in terms of their stimulation and activation. To computationally confirm this finding, we extend our study to also dock Hsp70 protein into the top‐scored receptors with the TKD sequence using Schrödinger Maestro software. The possible docking poses were then analyzed to find the binding energy using the HPEPDOCK Server (Table S2, Supporting Information). The comparison analysis showed no significant variations between the docking results of Hsp70 protein‐receptor and TKD peptide‐receptor complexes which is a proof of concept supporting TKD as the major binding site of Hsp70.

In contrast to the homodimer receptors, the biologically active form of CD94 is a heterodimer incorporated with either the inhibitory receptor NKG2A or the activating receptor NKG2C.^[^ ^24^ ^]^ Since there is no crystallographic structure of NKG2C alone or the complex CD94/NKG2C in the Protein Data Bank (PDB), the CD94/NKG2A complex was docked to the TKD ligand given the 77% similarity between the full sequences of NKG2A and NKG2C and the 92% identity of the extracellular domains of NKG2A and NKG2C, according to basic local alignment search tool (BLAST) results (Figure S1, Supporting Information).^[^ ^24^ ^]^ NKG2A alone which does not exist on the surface of NK cells exhibited a much weaker binding affinity than its complex with CD94, implying the dominant role of CD94 in interacting with TKD. Similar observations have been made for HLA‐E and CD94/NKG2A interactions, in that the CD94 subunit has a greater contribution (≈80%) than NKG2A in the complex relationship.^[^ ^25^ ^]^ Accordingly, the receptor complex has been named CD94/NKG2A/C in Figure 1 and Figure 3, Tables S1,S2, and Figure S3 (Supporting information).

### 2.2 Phenotypic Characterization of Primary NK Cells After Stimulation with IL‐2 and TKD Peptide

After docking, we first focused on narrowing the list to the top‐scored receptors for which cell surface expression quantification upon TKD stimulation was helpful. Therefore, the phenotypic characteristics of unstimulated NK cells, isolated from the peripheral blood of at least five healthy volunteers, were compared to NK cells stimulated with low‐dose IL‐2 (100 IU mL^−1^) alone or in combination with TKD peptide (2 µg mL^−1^) for 3 days at 37 °C. The data are expressed as fold change in the percentages and mean fluorescence intensity (MFI) values for the IL‐2 versus IL‐2/TKD stimulated cells (**Table** **1**). The dot plots showing the gating strategy for the immunophenotyping of the CD3^−^CD56^+^ NK cell subpopulations are illustrated in Figure S2 (Supporting Information).

**Table 1:** Frequency and expression density of activating and inhibitory NK cell receptors after stimulation with IL‐2 and IL‐2/TKD. Cell surface receptors of peripheral blood mononuclear cells (PBMCs)‐derived NK cells, kept either unstimulated (Unst) or stimulated (St) with IL‐2 (100 IU ml^−1^) or IL‐2/TKD (2 µg mL^−1^) for 3 days at 37 °C, were assessed by multiparameter flow cytometry using fluorochrome‐labeled monoclonal antibodies (mAbs). The data are expressed as mean percentages of positively stained cells (*n* ≥ 5 independent donors) and categorized as 0–5% (‐), 5.1‐24.9% (+), 25–49.9% (++), 50–74.9% (+++), and 75–100% (+++). The positivity and fold change in mean fluorescence intensity (MFI) values of IL‐2/TKD‐St NK cells were compared to IL‐2‐St NK cells. Levels of significance are shown as non‐significant (ns), ^*^ *p* ≤ 0.05, ^**^ *p* ≤ 0.01, ^***^ *p* ≤ 0.001.

Receptors	NK Cells			*p* Value	MFI Fold Change
	Unst	IL‐2‐St	IL‐2/TKD‐St	IL‐2 vs IL‐2/TKD	
NTB‐A (SLAMF6)	++++	++++	++++	ns	1.2
NKp30 (CD337)	+	+	+	ns	_
NKG2D (CD314)	++	+++	+++	ns	_
CD94	++	++	+++	*	1.2
CD69	+	++	+++	*	1.1
KIR2DS4	+	+	+	ns	_
FcγR (CD16)	++++	++++	++++	ns	0.9
NKp46 (CD335)	++	+++	+++	ns	_
KIR2DL4	+	+	++	ns	_
NKG2A (CD159a)	++	++	++	ns	_
NKp44 (CD336)	+	++	++	ns	1.1
DNAM‐1 (CD226)	++++	++++	++++	ns	_
OX40 (CD134)	‐	+	++	**	1.3
LAMP‐1 (CD107a)	‐	‐	+	***	1.6
PD‐1 (CD279)	‐	‐	‐	ns	_
NKG2C (CD159c)	+	+	+	ns	_

NK cell activation and the induction of cytotoxic programming against targets after interaction with TSAs can be triggered by various activating receptors and co‐receptors.^[^ ^7^ ^]^ As shown in Table 1, a comparison of common receptor expression reveals the engagement of NKG2D, CD69, NKp46, NKp44, and OX40 in response to IL‐2 stimulation, as has been previously reported.^[^ ^26^ ^]^ However, the proportions of NK cells expressing NKG2D, NKp46, and NKp44 and their expression level after stimulation with IL‐2 alone or IL‐2/TKD were not significantly different. This indicates that IL‐2 is responsible for upregulating the expression of NKG2D, NKp46, and NKp44. In contrast, expression of CD94 and CD69 and the lysosome‐associated membrane glycoprotein 1 (LAMP‐1; *p* ≤ 0.001) was significantly higher after IL‐2/TKD stimulation compared to IL‐2 alone, indicating that TKD has an additional stimulatory effect on NK cells. The surface exposure of LAMP‐1, a major component of late endosomes and lysosomes, implies NK cell degranulation following TKD stimulation and thus serves as a predictive marker for NK cell functionality.^[^ ^27^ ^]^ Moreover, the percentage of cells expressing the immune checkpoint modulator OX40 in IL‐2/TKD stimulated NK cells was significantly higher than their IL‐2 stimulated counterparts. It has been illustrated that OX40 is a co‐stimulatory receptor that is upregulated by pro‐inflammatory cytokines (such as IL‐2) on activated T cells or monocytes in a co‐culture.^[^ ^26a^ ^]^ Supplementary to IL‐2, our case showed the enhanced impact of TKD on OX40 expression. Similarly, the density of NTB‐A co‐stimulatory receptors increased upon IL‐2/TKD priming.

The molecular docking and immunophenotyping data reveal that NK cell activation by TKD correlates with an increase in the proportion of cells expressing CD94 and CD69 and the expression density (*p* < 0.05, 1.2‐fold; *p* < 0.05, 1.1‐fold, respectively). This phenomenon is in line with the docking performance of TKD, as CD94 and CD69 are ranked among our top five receptors (i.e., NTB‐A, NKp30, NKG2D, CD94, and CD69). Like NKG2D, the presence of TKD had no significant effect on the expression of NKp30. The density of NTB‐A expression, a costimulatory receptor that synergizes with NKp46 and NKG2D to amplify the NK cell activity and is required for an efficient degranulation of NKG2D positive NK cells against virus‐infected cells,^[^ ^28^ ^]^ was 1.2 fold higher on NK cells after stimulation with IL‐2/TKD compared to IL‐2 alone. These findings confirm previous studies reporting an upregulated expression of CD94 and CD69 on IL‐2/TKD‐activated NK cells in a murine glioblastoma model^[^ ^29^ ^]^ and in patients with advanced non‐small cell lung cancer (NSCLC).^[^ ^30^ ^]^

Given the reported inability of scrambled peptides to stimulate NK cell activity,^[^ ^31^ ^]^ we analyzed the binding affinities of the original TKD peptide to NK cell receptor and compared it to that of scrambled peptides. For this, the scrambled peptides were modeled by Swiss‐PDBViewer (SPDBV) software (Figure S3A, Supporting Information) which were then docked to the CD94/NKG2A/C receptor complexes. The results showed that the order of the amino acids in TKD is essential for docking since the scrambled peptides showed much lower binding affinities (Figure S3B, Supporting Information). This quantitative assessment highlights the specificity of the TKD sequence, showing the best interaction with the active site of the CD94 receptor complex. Therefore, the scrambled were excluded from further in silico and in vitro experiments.

### 2.3 Binding Potential of Hsp70 Protein to the NK Cell Receptors CD94 and CD69

**Figure 2:**
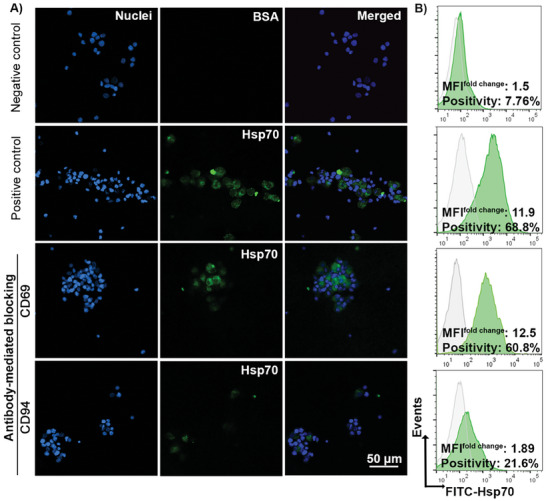
The role of CD69 and CD94 receptors in Hsp70 binding to NK cells after IL‐2/TKD stimulation. PBMC‐derived NK cells were stimulated with IL‐2/TKD for 3 days at 37 °C. The binding of FITC‐Hsp70 (40 µg/mL) to NK cells was investigated before and after mAb‐mediated blocking of the CD94 and CD69. A) The binding of FITC‐Hsp70 to the NK cell surface visualized by fluorescence microscopy. FITC‐BSA (bovine serum albumin) was used as a negative control. Nuclei were stained with DAPI (blue). The scale bar is 50 µm. B) Flow cytometric quantification of the bound FITC‐Hsp70 to the cell surface (green histograms). Data are expressed as positivity and MFI fold change compared to unstained control cells (gray histograms).

Based on the observed IL‐2/TKD‐induced increase in the proportion of CD69^+^ and CD94^+^ NK cells, we evaluated whether the communication of these receptors with the TKD binding site of Hsp70 protein could be blocked by CD69 and CD94 mAbs. For these studies, the enhanced expression of CD69 and CD94 after IL‐2/TKD stimulation was re‐confirmed by fluorescence imaging and flow cytometry (Figure S4, Supporting Information). As expected, the FITC‐Hsp70 protein, but not FITC‐BSA (bovine serum albumin), bound to IL‐2/TKD stimulated NK cells, expressing high intensities of CD94 and CD69 expression (**Figure** **2**A). Incubation of IL‐2/TKD stimulated NK cells with a CD94 mAb prior to co‐incubation with FITC‐Hsp70 protein markedly reduced the binding capacity, whereas incubation with a CD69 mAb had no effect on FITC‐Hsp70 binding (Figure 2A). The quantification of the results by flow cytometry verified the notable decline in FITC‐Hsp70 binding to the CD94 receptor after antibody‐mediated blocking, whereas the protein binding capacity to CD69 remained almost unchanged (Figure 2B). Receptor blocking with unlabeled and labeled CD69 and CD94 mAbs was also confirmed by flow cytometry (Figure S4, Supporting Information).

### 2.4 Molecular Dynamics Simulation (MDS)

**Figure 3:**
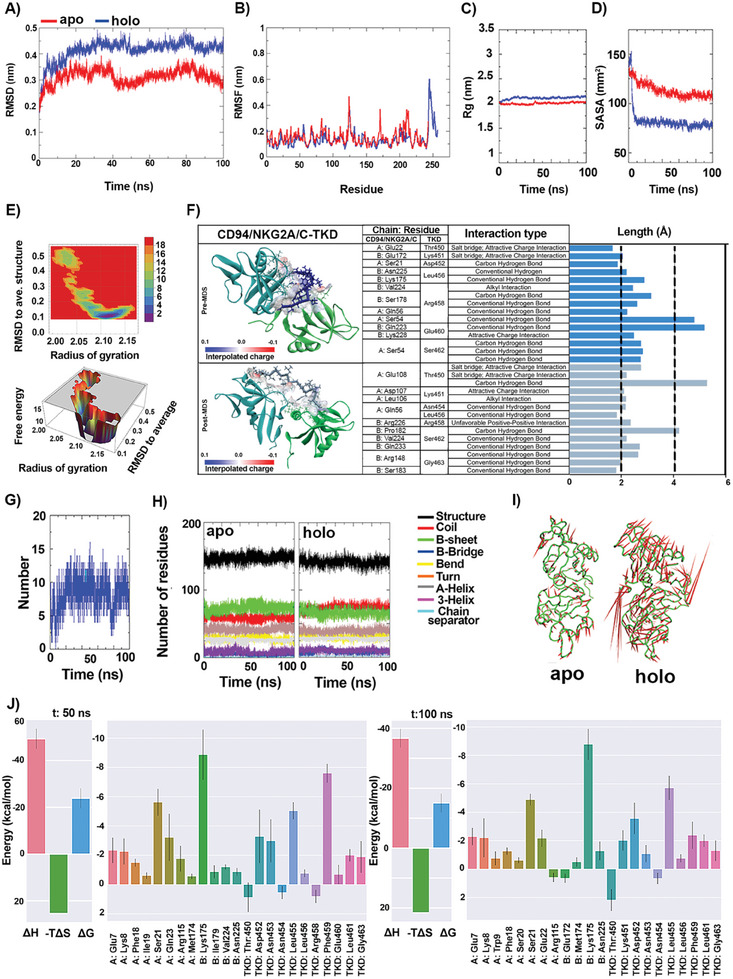
Molecular dynamics simulation. The complex of TKD peptide with CD94/NKG2A/C protein receptor was analyzed by molecular dynamics simulation (MDS). For this, the apo (receptor without ligand) and holo (receptor‐TKD complex) structures were put into a 100‐ns simulation time using GROMACS. A) The root mean square deviation (RMSD) values (nm), B) The root mean square fluctuation (RMSF) values (nm), C) The radius of gyration (Rg) values (nm), and D) Solvent accessible surface area (SASA) values (nm) plotted against time (ns) for apo (red) and holo (blue) structures. E) The 2D and 3D free energy landscape (FEL) diagrams of the holo receptor were depicted as a function of Rg and RMSD. F) The schematic representations (3D) of binding interactions (including bond types and length (A°) between the receptor active site and TKD residues in pre‐MDS (Cyan: CD94; Light green: NKG2A/C; Dark blue: TKD) and post‐MDS (Cyan: CD94; Light green: NKG2A/C; Light blue: TKD) complexes. The chains A and B refer to CD94 and NKG2A/C, respectively. G) Hydrogen bonds (H‐bonds) profile of holo form during MDS at 100 ns. H) Dictionary of the secondary structure of proteins (DSSP) presented as a function of time for apo and holo‐structures. I) Porcupine plots of apo and holo‐structures obtained by principal component analysis (PCA). J) The binding free energies (ΔG) between the residues interacting in holo‐structure calculated by molecular mechanics‐generalized born surface area (MM/GBSA) using interaction entropy approximation of last 10 frames at 50 ns and 100 ns simulation. The letters A and B refer to CD94 and NKG2A/C, respectively.

On the basis of molecular docking and immunophenotyping data, a series of computational techniques were used to check the detailed mode of interactions between protein receptor and ligand (either the full protein or its specific residues for binding) at the atomic level.^[^ ^21^ ^]^ To reach this aim, the best‐docked pose of CD94/NKG2A/C receptor in apo (protein receptor without ligand) and holo (protein receptor complexed with TKD or Hsp70) forms were submitted to MDS for a 100 nanoseconds (ns)‐time scale using Groningen Machine for Chemical Simulations (GROMACS) under periodic boundary conditions (PBC) (**Figure** **3**; Figure S5, Supporting Information).

MDS parameters such as root mean square deviation (RMSD) and root mean square fluctuations (RMSF) quantitatively evaluate the time‐dependent dynamic behavior of the complexes and protein residue flexibility in an aqueous solution, respectively. These parameters are indicative of the conformational stability of the systems under study.^[^ ^32^ ^]^ In the comparing A‐D panels of Figure 3 and Figure S5 (Supporting Information), the red line represents the RMSD/RMSF of the apo form, and the blue line refers to the holo form of the receptor. The computed RMSD for CD94/NKG2A/C reveals that the TKD binding in the active site could stabilize the complex, leading to good equilibration and a lower deviation (Figure 3). A comparable RMSD profile was obtained after Hsp70 binding to the receptor (Figure S5, Supporting Information) However, the RMSD trajectory of the complexes had a higher degree of conformational changes than the free receptor during the simulation. A jump observed within the first 5–10 ns is the consequence of the relaxation of the initial conformation.

To understand the dynamic behavior in more detail, the time‐averaged RMSF of protein receptors in apo and holo forms were plotted to visualize the individual residue flexibility/mobility (Figure 3B; Figure S5B, Supporting Information). As indicated by the RMSF results, most residues had RMSF values of less than 0.3 nm in the case of both TKD (Figure 3B) and Hsp70 complexes (Figure S5B, Supporting Information). The ligand‐bound receptor exhibited fewer residual fluctuations, but with somewhat different frequencies, particularly in more flexible regions with innately increased fluctuations. The sharp changes at the beginning and end of the curve result from the mobility of the N‐ and C‐termini of proteins. Furthermore, the radius of gyration (Rg) analysis was applied to reveal the compactness degree or folding properties of unligated and ligated receptors over time.^[^ ^33^ ^]^ Almost no effect of TKD (Figure 3C) and Hsp70 (Figure S5C, Supporting Information) on the compactness degree and rigidity of the CD94/NKG2A/C structure can be deduced from the slight difference of Rg rate between the receptor complex and the free form. However, the linear trend of Rg without deviations showed the conformational stability of the complex with TKD or Hsp70 along the simulation time. In addition to Rg, the solvent accessibility surface area (SASA) was used to evaluate the protein change after exposure to the solvents, thereby examining protein hydrophobicity.^[^ ^34^ ^]^ At the beginning of the simulation, the decreased SASA value of the holo form with TKD versus the apo forms indicates the folding state and compression of protein volume after ligation, which was then almost constant until the end of the simulation period (Figure 3D). The SASA value was higher for theHsp70‐receptor complex due to the protein expansion, showing its flexible nature than the truncated TKD‐receptor complex (Figure S3D, Supporting Information). As expected, the stable trajectories of SASA and Rg, to some extent at least, show quantitatively the steady binding state of the ligand‐receptor complexes over the entire phase of the 100 ns simulation. The different parameters applied in the MDS study, such as RMDS, RMSF, RG, and SASA, evidence a computational foundation that ligation of Hsp70 via its TKD sequence leads to a better stability of the CD94/NKG2A/C than the free receptor. Interestingly, TKD alone could confer almost similar stabilization to CD94/NKG2A/C upon binding in comparison with Hsp70.

The free energy landscapes (FEL) were also calculated to find the most stable conformation with the lowest binding free energy.^[^ ^35^ ^]^ The 2D and 3D FEL plots from MDS are shown in Figure 3E and Figure S5E (Supporting Information), using the RMSF‐embedded‐RMSD and Rg of the backbone as reaction coordinates. The plot shows a moderate distribution of complex conformations with one minimal energy cluster around which most structural conformations are concentrated. The most stable conformer was found at ≈76 and 85 ns of MDS for TKD‐ and Hsp70‐receptor complexes respectively, as indicated by the darkest blue regions with the lowest Gibbs free energy. The number and type of intramolecular bonding networks formed during MDS can also predict the stability of the ligand‐receptor complex, giving a clear picture of how the protein changes in terms of flexibility and shape.^[^ ^33^ ^]^ Because of this, the receptor‐ligand complex in pre‐ and post‐MDS states was visualized and studied by the Discovery Studio Visualizer program (Figure 3F). The most stable conformation identified from FEL analysis was extracted for post‐MD interaction analysis. A comparison was made with the interactions deduced from the pre‐MD docking results. From a comparative perspective, the residue pattern of the CD94/NKG2A/C involved in the interaction differs between the pre‐ and post‐MDS states, with glutamine (Gln) 56 being the only common residue. The same pattern was observed for the Hsp70‐CD94/NKG2A/C complex, as the interacting residues in the binding site changed completely during the MDS with the exception of tyrosine (Tyr) 83 (Figure S5F, Supporting Information). In essence, as the simulation proceeds, various residues of the active site are recruited to form the binding pocket. The predominant intermolecular contacts in both conformations are hydrogen bonds (H‐bonds), which are relatively shorter and hence stronger in the post‐MDS complex. Supplementary, an H‐bond analysis was carried out during the simulation. The full Hsp70 protein formed 39 H‐bonds with the residues of the CD94/NKG2A/C receptor (Figure S5G, Supporting Information). As seen in Figure 3G, TKD is involved in the formation of 1 to 16 bonds which is sufficient for a strong binding inside the active site pocket of the receptor.

A compelling aspect of our study lies in the analysis of the secondary structure of the receptor in its apo and holo forms (Figure 3H). The Dictionary of Protein Secondary Structure (DSSP) is the most common method to identify and assign the secondary structure elements (such as α‐helices, β‐sheets, and others) of each protein residue throughout the MD trajectory and thereby determine how they fluctuate and change over time.^[^ ^36^ ^]^ DSSP appropriately predicts the dynamic behavior of the protein by analyzing the backbone hydrogen bonding patterns and dihedral angles of the protein structure.^[^ ^37^ ^]^ Comparing the apo and holo forms throughout the MDS revealed minimal changes in most secondary structure elements, except for the β‐sheet and coil regions (Figure 3H). This analysis suggests that the TKD binding at the receptor active site is intricately linked to conformational stability, and is accompanied by alterations in folding and secondary structure elements. In contrast to TKD, the Hsp70 ligation induced remarkable changes in the secondary structure of the receptor which can be associated with more binding sites and stabilization of the receptor (Figure S3H, Supporting Information).

Principal component analysis (PCA) determines essential conformational changes and complex motions of biomolecules in MDS by projecting the data set into a lower‐dimensional subspace. Alignment of the trajectory snapshots to a common reference structure enables PCA to focus on intrinsic motions by analyzing the eigenvectors (principal components) and eigenvalues of the covariance matrix. The eigenvectors facilitate the visualization and interpretation of collective motions via which further insight can be gained about biomolecule dynamic modes, function, and potential allosteric effects.^[^ ^35^, ^38^ ^]^ For PCA in this study, a porcupine plot was drawn to visualize the first and second principal modes representing the essential movements of protein domains (Figure 3I; Figure S5I, Supporting Information). The analysis displays the distinct directions, and the magnitudes of the spines in the TKD‐CD94/NKG2A/C complex, compared to the free receptor, indicating explicit conformational changes induced by TKD and Hsp70 binding. PCA analysis effectively reduced the dimensionality of the molecular dynamics snapshots and led to cluster formation. Interestingly, our previous analyses suggest that these conformational changes may contribute to the overall stability of the complex.

As is widely acknowledged, docking algorithms possess inherent limitations in accurately representing the intricate details of biomolecular interactions. To address this issue and gain a more comprehensive viewpoint of the stability and energetics of protein‐ligand complexes, we implemented the molecular mechanics with the generalized Born surface area (MM/GBSA) method. This approach harnesses the detailed information from MDS trajectories to calculate the present binding free energy in the simulated complex. It therefore serves as a powerful tool to either corroborate and validate our docking results or identify instances where they may not hold true.^[^ ^33^, ^38^ ^]^ For this, the energies were obtained by taking ten snapshots from the last 50 and 100 ns trajectory periods. Regarding the TKD‐receptor complex, the negativity of the free energy and its slight difference (ΔG) between 50 ns (−22 kcal mol^−1^) and 100 ns (−15 kcal mol^−1^) denote sufficient free energy in support of a favorable binding affinity for a stable complex (Figure 3J). Similarly, the binding free energies of Hsp70 to CD94/NKG2A/C at snapshots of 50 ns (−34 kcal mol^−1^) and 100 ns (−43 kcal mol^−1^) confirm the docking results, suggesting strong binding between Hsp70 and the receptor. In addition, the discrepancy in the ΔG values between TKD and Hsp70 could possibly be due to their different amino acid lengths (Figure S5J, Supporting Information). In addition, the energy contributions per residue reveal that at both 50 ns and 100 ns of the simulation time, Serine (Ser) 21 (CD94), Lysine (Lys) 175 (NKG2A/C), and Phenylalanine (Phe) 459 (TKD) are the core functional amino acids with the highest binding energy (Figure 3J). In the case of Hsp70, it seems that Valine (Val) 224 (belongs to NKG2A/C) and Val 409 (belongs to Hsp70) play significant roles in the interaction between Hsp70 and CD94/NKG2A/C, exerting the most substantial impact on their binding (Figure S5J, Supporting Information). Collectively, the results provide a deeper insight into the binding properties of the TKD compared to the full‐length Hsp70, thereby enabling a better appreciation of the effect of TKD on the CD94/NKG2A/C receptor structure after ligation. These results are in line with the molecular docking and dynamics records and the observed biological studies that have been published previously.^[^ ^31^, ^39^ ^]^

### 2.5 CRISPR/Cas9‐Mediated CD94 Gene Knockout in Primary NK Cells

**Figure 4:**
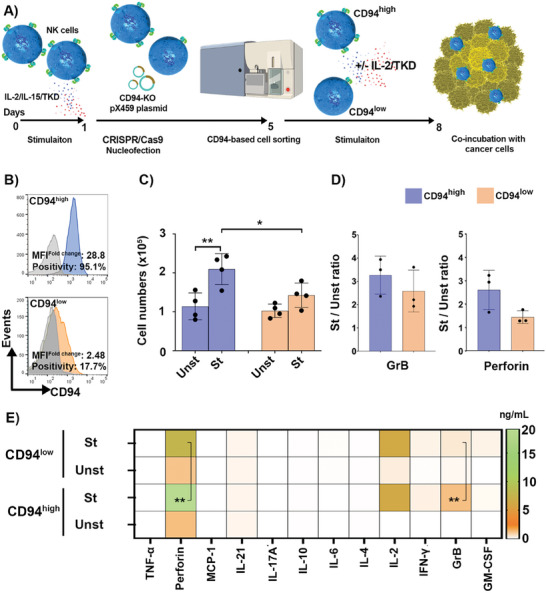
Functional features of two subpopulations of NK cells expressing distinct levels of CD94 receptor after stimulation with IL‐2/TKD. A) Schematic overview of the in vitro experiments. Cells pre‐treated with IL‐2/IL‐15/TKD were subjected to CRISPR/Cas9 nucleofection using CD94 knockout (KO) pX459 plasmid. After 4 days, the cells were sorted based on the CD94 expression. CD94^high^ and CD94^low^ NK cell populations were stimulated with IL‐2/TKD for 3 days at 37 °C. The functional characteristics of stimulated CD94^high^ and CD94^low^ NK cell populations were compared to those of Unst NK cells, either in mono or in co‐culture with cancer cells. B) Representative flow cytometric analysis of CD94 expression on sorted NK cells at day 3 after IL‐2/TKD stimulation (blue/orange histograms). Isotype‐matched mAb was used as a negative control (gray histograms). Data are shown as positivity percentage and MFI fold change. The fold change was assessed by dividing the expression value of the CD94 positive cells by the values generated for isotype‐treated control cells. C) Expansion potential of sorted NK cells. Cell numbers were determined based on viable cell counts after 3 days of incubation of NK cells (2 × 10^5^ cells well^−1^) in cell culture medium with/without IL‐2/TKD (*n* ≥ 4 independent donors, triplicate mean ± SD). D) The positivity ratio of IL‐2/TKD‐St to Unst NK cells for intracellular perforin and GrB contents (*n* ≥ 3 independent donors, triplicate mean ± SD). E) Multiplex cytokine/chemokine levels quantified by flow cytometry in the supernatant media of St compared to Unst NK cells (*n* ≥ 3 independent donors, triplicate mean). Levels of significance are shown as ^*^ *p* ≤ 0.05, **p ≤ 0.01.

To further interrogate the role of CD94 in NK cell interactions with Hsp70 via the TKD interaction site, CRISPR/Cas9‐based gene editing was used to knockout the CD94 gene (CD94‐KO) in activated NK cells, according to the scheme in **Figure** **4**A. To achieve a high transfection efficiency, the CRISPR/Cas9 plasmid was nucleofected into NK cells^[^ ^40^ ^]^ after incubation with IL‐2/IL‐15/TKD which drastically enhances NK cell proliferation (Figure S6, Supporting Information).^[^ ^4a^ ^]^ On day 5, the nucleofected cells were sorted into CD94^high^ and CD94^low^ populations by fluorescence‐activated cell sorting. The sorted NK cells were then subjected to flow cytometry to evaluate the expression level of CD94 after a 3‐day stimulation period with IL‐2/TKD. The stimulation of NK cells by cytokines enhances the expression of activating receptors on NK cells and the combination of IL‐2/TKD elevates the expression of CD94.^[^ ^13^, ^31^ ^]^ The proportion of CD94 positive cells in the CD94^high^ NK cell population was 95.1% and that of the CD94^low^ population was 17.7% indicating a successful knockout of CD94 after subsequent cell sorting (**Figure** **4**B). The potential for proliferation, lytic granule release, and specific cytokines/chemokines secretion by IL‐2/TKD activated CD94^high^ and CD94^low^ NK cell populations were compared in mono‐ and co‐cultures.

Previous studies revealed the importance of an increased expansion potential of NK cells ex vivo and their prolonged life span in vivo, although the antigenic/cytokine stimuli and the involved receptor mechanisms may also control proliferation and survival.^[^ ^41^ ^]^ Voss et al have previously reported on the involvement of ligated CD94 in the costimulation of NK cells and the augmented proliferative response to IL‐2 or IL‐15, particularly in the CD56^bright^ cell population.^[^ ^41a^ ^]^ In our study, CD94^high^ NK cells persisted at higher cell numbers than CD94^low^ NK cells over 3 days after IL‐2/TKD stimulation (*p* ≤ 0.05). The differences in the number of IL‐2/TKD stimulated (St) and unstimulated (Unst) NK cells are more significant in the CD94^high^ NK cell subset. This response might be driven by the CD94‐TKD interaction and promoted by IL‐2 (Figure 4C).

The activation status of effector cells can be characterized by elevated levels of effector molecules such as granzyme B (GrB) and the pore‐forming proteins (e.g., perforin and granulysin) in the cytoplasm.^[^ ^9^, ^42^ ^]^ Our results clearly indicate higher intracellular levels of GrB and perforin in IL‐2/TKD stimulated NK cells compared to unstimulated naïve NK cells (Figure 4D). The serine protease GrB is an essential constituent of the granules which serves as a marker for activated cytotoxic effector cells.^[^ ^43^ ^]^ Perforin serves as another potential marker of NK cell activation which is effectively involved in their lytic activity.^[^ ^43^ ^]^ The efficient trafficking of perforin to the lytic granules and the degranulation process of NK cells also depend on LAMP‐1.^[^ ^27^ ^]^ LAMP‐1 on the cell surface of NK cells is associated with the intracellular accumulation and the extracellular release of perforin, depending on the stimulation state.^[^ ^44^ ^]^ Also in our study, a similar trend was found for LAMP‐1 and lytic granules. The concentrations of GrB and perforin increased in the supernatant of IL‐2/TKD stimulated NK cells and were even higher in the sorted CD94^high^ population than the CD94^low^ NK cell population (**p < 0.01) (Figure 4E). When looking at the multiplex panel of immune biomarkers assayed in the supernatants, there were no significant differences in cytokine profiles between stimulated and unstimulated NK cells, except for IFN‐γ and granulocyte‐macrophage colony‐stimulating factor (GM‐CSF) (Figure 4E). However, these differences were independent of the CD94 expression. IFN‐γ is a secretory product of NK cells, known as an index of stimulation and immune responsiveness.^[^ ^41^, ^45^ ^]^ Incubation of NK cells with IL‐2/TKD induces IFN‐γ release, whereas IL‐2/TKD stimulation has no effect on CD3^+^ T cells.^[^ ^46^ ^]^ The secretion of multifunctional GM‐CSF together with other factors such as IFN‐γ is also linked to NK cell responses to antigen/cytokine stimulation. Overall, our findings provide additional insight into the capacity of TKD in combination with IL‐2 to significantly favor the potential for NK cell expansion, and cytokine and GrB release, especially in CD94^high^ NK cells.

### 2.6 Interaction of IL‐2/TKD Stimulated CD94^high^ and CD94^low^ NK Cells and Tumor Cells Expressing mHsp70

**Figure 5:**
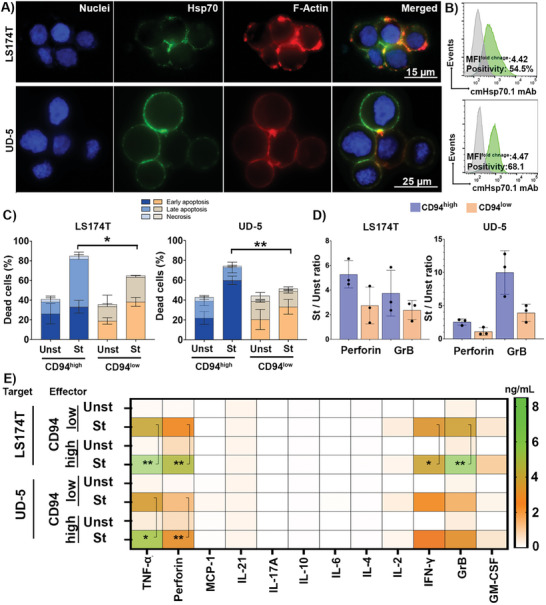
Interaction between mHsp70‐expressing cancer cells and IL‐2/TKD‐St CD94^high^ and CD94^Low^ NK cells via ligand‐receptor binding processes. A) Representative fluorescence images of LS174T and UD‐5 cells expressing high densities of membrane Hsp70 (mHsp70) using FITC‐cmHsp70.1 mAb (Green). Nuclei and F‐actin skeleton were stained with DAPI (Blue) and phalloidin (Red), respectively. The scale bars are 15 and 25 µm for LS174T and UD‐5, respectively. B) Representative flow cytometric measurement of mHsp70 expression using FITC‐cmHsp70.1 mAb (Green histograms). Isotype‐matched (Mouse FITC‐IgG1) mAb was used as negative control (Gray histograms). Data are shown as positivity percentage and MFI fold change. The fold change was assessed by dividing the expression value of the FITC‐cmHsp70.1‐treated cells by the values generated for isotype‐matched cells. C) Cancer cell apoptosis detected by Annexin V/PI assay. Prior to measurement, target cells (2 × 10^5^ cells well^−1^) were incubated with IL‐2/TKD‐St NK cells at a ratio of 1:1 for 8 h at 37 °C (*n* ≥ 3 independent donors, triplicate mean ± SD). D) The positivity ratio of IL‐2/TKD‐St to Unst NK cells for intracellular perforin and GrB contents after an 8‐h co‐incubation with cancer cells. E) Multiplex cytokine/chemokine levels quantified by flow cytometry in the supernatant media. For this, the cancer cells were co‐cultured with IL‐2/TKD‐St NK cells for 8 h. Levels of significance are shown as ^*^ *p* ≤ 0.05, ^**^ *p* ≤ 0.01.

The optimal response of NK cells to tumor targets is controlled by a fine balance between the receptor‐transduced inhibitory and activating signals towards specific stress ligands on tumor cells. After the interaction of the ligand with its corresponding receptor, activated NK cells kill target cells mainly through exocytosis of granules containing lytic proteins, hypersecretion of tumor necrosis factor (TNF) family, death ligands, effector molecules (e.g., IFN‐γ), and ADCC.^[^ ^9^ ^]^ Given the crucial role of receptor‐ligand crosstalk in immune cell functions and subsequent tumor cell fate, we monitored the impact of tumor‐expressed mHsp70 on the ability of IL‐2/TKD activated CD94^high^ and CD94^low^ NK cells to secrete cytokines and lyse cancer cells. The success of a receptor‐ligand interaction is tightly linked to receptor availability, as well as affinity for the target antigen and its expression profile on tumor target cells.^[^ ^3^ ^]^ Based on these principles, LS174T human colorectal adenocarcinoma and UD‐5 human squamous cell carcinoma cell lines were used as targets due to their high expression of mHsp70, as determined by fluorescence microscopy (**Figure** **5**A). Considering the shape of the cells outlined by F‐actin staining (red), the surface density of the mHsp70 was visualized in green using FITC‐labeled cmHsp70.1 mAb. Flow cytometric analysis of viable LS174T and UD‐5 tumor cells revealed mHsp70 expression on 54.5% and 68.1% of cells, with an MFI of 4.42 and 4.47, respectively (Figure 5B).

The cytolytic capacity of IL‐2/TKD stimulated CD94^high^, CD94^low^, and unstimulated NK cells against LS174T or UD‐5 target cells tumor cells was determined at effector to target cell ratio of 1:1 (E:T 1:1) over an 8‐h coincubation using a standard Annexin V/PI assay (Figure 5C). The gating strategy is outlined in Figure S7 (Supporting Information). Our findings revealed that ≈80% of tumor cells were killed by stimulated CD94^high^ NK cells, whereas only ≈40% of tumor cell death was detected after incubation with unstimulated NK cells. The killing capacity of IL‐2/TKD activated CD94^low^ NK cells was less pronounced than that of CD94^high^ NK cells, particularly when UD‐5 cells were used as target cells. This finding confirms the positive regulatory role of the CD94‐Hsp70 interaction on the lytic activity of NK cells. A tight contact between effector and target cells ensures the establishment of a functional immune synapse (IS), a dynamic structure that balances and integrates activating and inhibitory receptor signaling.^[^ ^8^ ^]^ The formation of the IS triggers the intracellular cytotoxic machinery of NK cells and a specific and efficient eradication of target cells.^[^ ^8^ ^]^ However, the progression of target cells through the apoptotic pathway triggered by CD94^high^ NK cells appears to be cancer cell line dependent. After an 8‐h co‐incubation of tumor target cells with IL‐2/TKD stimulated CD94^high^NK cells, LS174T cells were already in late apoptosis, whereas the majority of UD‐5 cells were in early apoptosis. CD94 in the context of NKG2A mediates inhibitory signals to NK cells whereas a combination with NKG2C mediates activating signaling. However, as shown by Kim et al.^[^ ^47^ ^]^ ligation of the inhibitory NK cell receptor has an inhibitory and at the same time may have a licensing effect on NK cells, whereas ligation of the activating NK cell receptor complex has an activating effect and at the same time may cause downward resetting of the educated NK cells.^[^ ^48^ ^]^ Therefore, the synergy between the activation and licensing of different ligands on tumor cells might be important for the function of NK cells. Previous results of our group have demonstrated that^[^ ^49^ ^]^ that tumor cells expressing Hsp70 in the absence of HLA‐E are killed best by IL‐2/TKD activated CD94^+^/NKG2A^+^ NK cells followed by tumor cells expressing both Hsp70 and HLA‐E. The lowest lysis is detected in tumor cells that neither express Hsp70 nor HLA‐E on their cell surface. A transfection experiment of different HLA‐E alleles into a tumor cell line with stable Hsp70 expression revealed a correlation between the amount of HLA‐E expression on the tumor target cells and the downregulation of lysis by IL‐2/TKD activated CD94^+^/NKG2A^+^ NK cells. These data indicate that the hierarchy and dominance of activating or inhibitory ligands determine the lytic activity of sIL‐2/TKD‐activated CD94^+^/NKG2A^+^ NK cells.

The capacity of activated NK cells to lyse tumor cells was associated with the intra‐ and extracellular levels of GrB and perforin in NK cells (Figure 5D). Comparing the ratios of lytic granules in IL‐2/TKD stimulated CD94^high^ and CD94^low^ NK cells to their unstimulated counterparts after co‐incubation with UD‐5 and LS174T cells revealed higher GrB and perforin levels CD94^high^ NK cells (Figure 5D). In agreement with the apoptosis results, perforin/GrB secretion was also associated with CD94 expression in activated NK cells. However, an apparent target cell‐associated difference in the concentration of GrB and perforin in the supernatant was observed, with higher levels being present when LS174T were used as targets, as compared to UD‐5 cells. This highlights the fact that although specific antigen expression on cancer cells is necessary for a desired interaction with NK cell receptors, antigen quality, and quantity have a determinative role in the successful formation of the IS and the resultant NK cell lytic response against cancer cells. The strength of the lytic hit by NK cells can control the death kinetics in target cells.^[^ ^50^ ^]^ Following antigen recognition and IS formation, perforin/GrB released into the intercellular space facilitates lytic granule‐mediated target cell DNA fragmentation and apoptosis. High levels of released perforin induce fast death accompanied by target cell osmotic lysis, whereas sub‐lethal levels regulate apoptosis of the targeted cells cooperatively with GrB.^[^ ^42^ ^]^ A quantitative study has previously reported that a minimum number of degranulation events are required for favorable perforin/GrB‐mediated cytotoxicity by NK cells, either in cell lines or primary cells, which allows NK cells to perform serial killing of multiple target cells.^[^ ^50^ ^]^ It should be noted that despite the complementary relationship between GrB and perforin, GrB can also initiate apoptosis in a perforin‐independent manner.^[^ ^51^ ^]^

Targeting mHsp70 expressing cancer cells by NK cells also triggers the secretion of inflammatory cytokines such as TNF‐α and IFN‐γ (Figure 5E). In addition to a differential release from stimulated and unstimulated NK cells, it is apparent that the CD94‐Hsp70 interplay is important for the hypersecretion of pro‐inflammatory cytokines, as seen in the cytokine levels in the supernatants of CD94^high^ and CD94^low^ NK cells. TNF‐α contributes to effective immunity against malignancy through death signal transduction by binding to tumor death receptors.^[^ ^52^ ^]^ TNF‐α can also regulate serial killing by NK cells by altering the expression pattern of molecules involved in target cell recognition and IS formation.^[^ ^8^ ^]^ TNF‐α can also augment NK cell differentiation and activation, as well as cytokine (e.g., IFN‐γ) production.^[^ ^52^ ^]^ Several studies have reported the synergistic and complementary apoptosis‐mediated anti‐tumor effects of IFN‐γ and TNF‐α.^[^ ^53^ ^]^ As proved, IFN‐γ can also induce an increase in the expression of Tumor Necrosis Factor Related Apoptosis Inducing Ligand (TRAIL) in an autocrine fashion, thereby accelerating TNF‐related apoptosis in cancer cells.^[^ ^53^ ^]^ Coincident with the dramatic increase in TNF‐α, IFN‐γ, and lytic granule production, stimulated CD94^high^ secreted more GM‐CSF than CD94^low^ NK cells. The production of cytokines such as GM‐CSF by NK cells can be partially regulated by differential surface receptor activation.^[^ ^41b^ ^]^ In accordance with this concept, our results suggest that the molecular mechanisms underlying cytokine production are likely dependent on CD94 and its ligation with Hsp70. This claim is more meaningful in the co‐culture of NK cells with LS174T cells than UD‐5. Taken together, our study evidence that the superior anti‐tumor activity of IL‐2/TKD stimulated NK cells may be largely associated with increased perforin/GrB degranulation and cytokine/chemokine secretion. In almost all conditions, the CD94‐Hsp70 interaction induced significant differences in immune responses against tumor cells in a target cell‐dependent manner. Target cell‐dependent responses may originate from cancer cell characteristics such as growth potential, immune resistance, and specifically, IS formation.

## 3 Conclusion

In summary, the selectivity and functional avidity of the Hsp70‐derived TKD peptide with specific receptor/s on primary NK cells have been interrogated using in silico and in vitro approaches. There is a positive correlation between TKD peptide binding and the surface expression level of several receptors, especially CD94. CD94 has also been shown to play a key role in recognizing mHsp70, most probably via the TKD binding site, on tumor cells and mediating NK cell lytic activities. Consolidation of the findings demonstrates that CD94 has a manifest task in Hsp70 recognition and possibly signal transduction pathways regarding NK cell stimulation and activation. Hence, elevated CD94 expression following cytokine/TKD stimulation can serve as a useful surrogate marker for determining the Hsp70‐induced reactivity of NK cells. Since the receptor‐ligand interaction is critical for appropriate immune control, our findings can offer valuable perspectives and insights for drug design and development.

## 4 Experimental Section

### Dataset

For peptide‐protein docking, the TKD sequence of TKDNNLLGRFELSG (amino acid numbers: 450–463) was extracted from the Hsp70 protein sequence (ID: 6K39) in the PDB. The PDB formatted file of NK cell receptors including NTB‐A, NKp30, NKG2D, CD94/NKG2A, CD69, KIR2DS4, CD16, 2B4, HLA‐DR, NKp46, KIR2DS2, KIR2DL4, NKG2A, NKP44, DNAM‐1, CD94, CD160, OX40, 4‐1BB, LAMP‐1, and PD‐1 were downloaded with the PDB codes of 2if7, 3noi, 4s0u, 3bdw, 1fm5, 3h8n, 1e4j, 2ptu, 1d6e, 6iap, 4n8v, 3wyr,3bdw, 1hkf, 6isb, 1b6e, 6ng9, 6okm, 6a3w, 5gv0, and 6umu, respectively. Both ligand and protein receptors were processed by adding polar hydrogens and removing heavy atoms, water molecules, and ions. Complete frames were then considered for ligand‐receptor interactions.

The sequences of NKG2C (accession numbers QSG30230.1) and NKG2A (accession numbers AAL65234.1) were obtained in the FASTA file from the NCBI database. The BLAST was used to compare the NKG2C and NKG2A sequences, followed by an additional alignment step by the CLC Sequence Viewer software (version 8). SPDBV (version 4.0.1) was applied to extract the scrambled peptides of TKDNNLLGRFELTG, LKDNNLLGRFELSG, and NGLTLKNDFSRLEG followed by minimization steps. The scrambled peptides were aligned for comparison with TKD using the CLC Sequence Viewer software.

### Molecular Docking

Molecular docking was undertaken using Schrödinger Maestro software (Maestro, Schrödinger, LLC, New York, NY, 2018). Briefly, the ligand and protein receptors were prepared via the Protein preparation wizard for optimizing hydrogen bonding patterns at pH 7.0 (using PROPKA) and energy minimization (using the Optimized Potentials for Liquid Simulations (OPLS)−2005 force field) with heavy atoms converging to 0.3 Å. A receptor grid box was defined via the Glide‐receptor grid generation tool in Maestro, and blind ligand‐receptor docking was then carried out in SP‐peptide docking mode. The binding affinity was determined in kcal/mol and sorted based on the higher negative values implying the best docking score. The predicted interactions were visualized and studied in terms of bond types and length in BIOVIA Discovery Studio software (2016). The peptide‐protein docking was also studied through the HPEPDOCK server to confirm binding affinity.

### MDS Analysis

To validate the docking results and investigate the interaction behavior of the NK cell receptor with ligand at the atomic level, MDS was performed using GROMACS software (version 2018.4). The apo and holo forms of receptor were taken as initial inputs for 100 ns simulations. The Assisted Model Building with Energy Refinement (AMBER)99SB force field was applied to parametrize the protein receptor and ligand. The water model of transferable intermolecular potential water molecules (TIP3P) was selected to solvate a cubic box with counterions to neutralize the system. The peptide/protein water system was subjected to PBC. The stimulation box was then energy‐minimized using the steepest descent algorithm, followed by two‐step equilibration. The initial 1 ns equilibration was performed at constant volume using a number‐volume‐temperature (NVT) ensemble with the temperature set at 310 K. The second one was equilibrated for 1 ns using a number‐pressure‐temperature (NPT) ensemble at 1 bar constant pressure with a coupling constant of 0.1 picosecond (ps) using Berendsen barostat retaining the pressure.^[^ ^54^ ^]^ The dynamics simulation process was controlled at constant temperature (310 K) and pressure (1 atm) with the Nose‐Hoover thermostat^[^ ^55^ ^]^ and Martyna Tobias‐Klein^[^ ^56^ ^]^ algorithms, respectively. The geometries were constrained with the SETTLE algorithm^[^ ^57^ ^]^ and the bond lengths were with Linear Constraint Solver (LINCS).^[^ ^58^ ^]^ Electrostatic interactions were calculated based on the Particle Mesh Ewald (PME) algorithm.^[^ ^59^ ^]^ The trajectories were generated every two femtoseconds (fs) and saved every 2 ps. The structural behavior and conformational changes were studied through post‐MDS analyses, including the RMSD, RMSF, Rg, SASA, and H‐bond, followed by FEL, PCA, and MM‐GBSA. The output visualization was created via Xmgrace, Discovery Studio, and Visual Molecular Dynamics (VMD) software.^[^ ^60^ ^]^

### NK Cell Isolation

PBMCs were isolated from the peripheral blood of various healthy donors via density gradient centrifugation. Anonymous collection and use of PBMCs from volunteers were in compliance with the Technical University of Munich ethics commission (Protocol code 2428/09). NK cell enrichment of isolated PBMCs was performed using NK Cell Isolation Kit (130‐092‐657; Miltenyi Biotec) which utilizes magnetic‐activated cell sorting (MACS).

### Cells and Cell Culture

The PBMC‐derived NK cells were primarily cultured in RPMI‐1640 supplemented with 10% v/v heat‐inactivated fetal bovine serum (FBS; Sigma‐Aldrich), 1% v/v antibiotics (100 IU/mL penicillin and 100 µg mL^−1^ streptomycin; Sigma‐Aldrich), 2 mM L‐glutamine (Sigma‐Aldrich), and 1 mM sodium pyruvate (Sigma‐Aldrich). The human colorectal adenocarcinoma cell line LS174T (ATCC CL‐188; ATCC, Manassas, VA, USA) and human head and neck squamous cell carcinoma UD‐SSC 5, (UD‐5; Clinic of Otolaryngology, Düsseldorf, Germany) were cultured in supplemented high glucose (4 g L^−1^ glucose, Sigma‐Aldrich) Dulbecco's Eagle's Minimum Essential Medium (DMEM). The cells were incubated under controlled conditions at 37 ˚C with 95% v/v relative humidity, and 5% v/v CO_2_. All cell lines were regularly tested, and shown to be negative for mycoplasma test, and their viability was checked before each experiment using a trypan blue exclusion assay.

### NK Cell Stimulation

For ex vivo stimulation, primary NK cells (5 × 10^6^ cells mL^−1^) were incubated in a cell culture medium containing low dose IL‐2 (100 IU mL^−1^; Chiron) alone or in combination with TKD peptide (2 µg/mL; multimmune GmbH) for 3 days at 37 ˚C, as previously described.^[^ ^4a^ ^]^ IL‐15 (5 ng mL^−1^; PeproTech) was also applied for the optimal stimulation level for nucleofection.

### NK Cell Immunophenotyping

A flow cytometric analysis of PBMC‐derived NK cells was carried out using fluorescence‐conjugated mAbs including VioBlue‐conjugated anti‐CD3 (clone REA613; Miltenyi Biotec), APC‐Vio‐conjugated anti‐CD56 (clone REA196; Miltenyi Biotec), VioBright FITC‐conjugated anti‐NKG2A (clone REA110; Miltenyi Biotec), PE‐conjugated anti‐PD1 (clone REA1165; Miltenyi Biotec), PE‐Vio 770/APC‐conjugated anti‐CD94 (clone REA113; Miltenyi Biotec/clone HP‐3B1; Beckman Coulter), APC‐conjugated anti‐NKG2C (clone REA205; Miltenyi Biotec), FITC‐conjugated anti‐LAMP‐1 (clone REA792; Miltenyi Biotec), PE‐conjugated anti‐NKG2D (clone REA797; Miltenyi Biotec), PE‐Vio 770‐conjugated anti‐NKp44 (clone REA1163; Miltenyi Biotec), APC‐conjugated anti‐CD69 (clone REA824; Miltenyi Biotec), FITC‐conjugated anti‐KIR2DS4 (clone REA860; Miltenyi Biotec), PE‐conjugated anti‐NKp30 (clone REA823; Miltenyi Biotec), PE‐Vio 770‐conjugated anti‐NKp46 (clone REA808; Miltenyi Biotec), APC‐conjugated anti‐OX40 (clone REA621; Miltenyi Biotec), FITC/PE‐conjugated anti‐CD16 (clone REA423; Miltenyi Biotec/clone 3G8; BD), PE‐conjugated anti‐NTB‐A (clone REA339; Miltenyi Biotec), FITC‐conjugated anti‐KIR2DL4 (clone REA860; Miltenyi Biotec), and APC‐conjugated anti‐DNAM‐1 (clone REA1040; Miltenyi Biotec). The expression levels of cell surface receptors on propidium‐iodide (PI)‐negative viable cells were quantified by MACSQuant Analyzer 9 flow cytometer (Miltenyi Biotec), and the acquired data were analyzed using FlowJo software (version 10.8). The positivity percentage of stained cells and MFI were calculated against the background obtained with isotype‐matched control mAbs.

### Receptor Detection and Blocking Study

Fluorescence‐based studies such as fluorescence microscopy and flow cytometry were performed to analyze the binding affinity of the full‐length Hsp70 protein to the NK cell receptors before and after an antibody‐mediated blocking experiment. Prior to the study, the PBMC‐derived NK cells were stimulated with IL‐2/TKD to induce an upregulation of activating receptors (CD69 and CD94). Stimulated NK cells (2‐3 × 10^5^ cells well^−1^) were incubated with either CD69 (clone REA824; Miltenyi Biotec) or CD94 (clone HP‐3B1; Beckman Coulter) blocking mAbs in phosphate‐buffered saline (PBS) containing 10% w/v FBS for 15 min on ice. An isotype‐matched mAb was used as a negative control. After two washing steps with ice‐cold PBS, the cells were treated with FITC‐labeled Hsp70 protein (40 µg mL^−1^) for 15 min on ice followed by an additional washing step. BSA was tested under similar conditions to detect nonspecific protein binding. For imaging, the cells were fixed (Invitrogen) and then embedded in VECTASHIELD Hardset Antifade Mounting Medium with DAPI (Vector Laboratories). Images were acquired on a Leica DMi8 Thunder Imager System equipped with a 63x oil objective. To quantify the results, the non‐fixed cells were used for flow cytometry analysis.

### Ex Vivo Expansion of NK Cells

The expansion potential of PBMC‐derived NK cells was assessed to determine the optimum condition for nucleofection. For this, cells were seeded in a 12‐well plate (2 × 10^6^ cells well^−1^) under different stimulation conditions for 3 days: 1) medium without stimuli as control;2) IL‐2; 3) IL‐2/TKD; 4) IL‐2/IL‐15; 5) IL‐2/IL‐15/TKD. The persistence and expansion of stimulated NK cells were calculated by the viable cell counting method. Data were normalized to the time point of cell seeding and presented as the fold change mean of independent triplicate counting of *n* ≥ 4 individual healthy donors.

### CRISPR/Cas9–Mediated Gene Deletion of CD94 in NK cells

The single guide RNAs (sgRNAs) forward and reverse oligonucleotides were designed to target CD94 using the online CRISPick tool. The sgRNA targeting the desired sequences were as follows: #1 sgRNA forward: 5′‐CACCGCCAACGTAGACATCAACGAA‐3′; #1 sgRNA reverse: 5′‐AAACTTCGTTGATGTCTACGTTGGC‐3′; #2 sgRNA forward: 5′‐CACCGACATAGAACTCCAGAAAGGT‐3′; #2 sgRNA reverse: 5′‐AAACACCTTTCTGGAGTTCTATGTC‐3′. The sgRNA‐encoding sequences were annealed and ligated into the Cas9 vector pX459 (pSpCas9(BB)−2A‐Puro; Addgene plasmid ID:62 988) to construct the knockout plasmid. NK cells (10^6^ cells/Nucleocuvette strip) were nucleofected with pX459 using an Amaxa P3 Primary Cell 4D‐Nucleofecter X kit (V4XP‐3032; Lonza) and EK‐100 program on a 4D‐Nucleofector unit (Lonza). Cells were pre‐treated with IL‐2, IL‐15, and TKD to achieve optimal efficiency. After transfection, the cells were kept in an incubator for four days and then sorted according to CD94 expression by flow cytometry (BD FACSAria Fusion) and directly collected into an Eppendorf tube containing supplemented RPMI‐1640 medium for further experiments.

### In Vitro Expansion of Sorted NK Cells

Sorted CD94^high^ and CD94^low^ NK cells (2 × 10^5^ cells well^−1^) were seeded in a 96‐well U‐bottom plate and stimulated with IL‐2/TKD for 3 days. Unstimulated cells acted as comparators in the experiments. Cell numbers were determined based on viable cell counts. Data were presented as a mean of independent triplicate counting of *n* ≥ 4 individual healthy donors on day 3 after stimulation.

### Intracellular GrB and Perforin Expression Analysis

Intracellular expression of GrB and perforin in NK cells was determined after either a single culture or an 8‐h co‐culture with LS174T and UD‐5 target cells. Prior to the experiment, the NK cells were left unstimulated or stimulated with IL‐2/TKD. For the experiment, cells were fixed by adding 200 µL well^−1^ of the fixation solution (Invitrogen) for 1 h at room temperature (RT), washed twice with permeabilization solution, and incubated for a further 1 h with 100 µL well^−1^ of 1x permeabilization solution (Invitrogen) containing anti‐GrB (clone REA226; Miltenyi Biotec) and anti‐perforin (clone REA1061; Miltenyi Biotec) mAbs. The samples were then washed with flow cytometry buffer (PBS containing 10% v/v FBS), and the expression levels of GrB and perforin were analyzed by flow cytometry. At least 2 × 10^4^ cells were acquired for each sample, from which MFI was determined using FlowJo software.

### Multiplex Cytokine Analysis

Extracellular release of inflammatory cytokines (e.g., GM‐CSF, IFN‐γ, IL‐2, IL‐4, IL‐6, IL‐10, IL‐17A, IL‐21, MCP‐1, and TNF‐α) and cytotoxic markers (e.g., Perforin, GrB) were quantitatively measured in the cell culture medium of a single cultured or 8‐h co‐cultured NK cells with LS174T and UD‐5 cells. Prior to the experiment, the NK cells were left unstimulated or stimulated with IL‐2/TKD. According to the MACSPlex Cytotoxic T/NK cell kit (Miltenyi Biotec) instructions, supernatants were incubated with antibody‐coated MACSPlex beads, followed by the addition of biotinylated detection reagents. Formed sandwich complexes were analyzed on the flow cytometer. The analyte concentrations in each sample were estimated based on the standard curve generated from known quantities of analytes under the same conditions.

### Quantification of mHsp70 Expression

LS174T and UD‐5 cells (2 × 10^5^ cells) were suspended in flow cytometry buffer containing either FITC‐labeled cmHsp70.1 mAb (20 µg mL^−1^, multimmune GmbH) or respective isotype control (mouse IgG1 FITC; BD Biosciences) for 30 min on ice in the dark. Cells were washed twice, followed by re‐suspension in a flow cytometry buffer with 1 µg mL^−1^ of PI.^[^ ^61^ ^]^ Samples were acquired by flow cytometry and analyzed using FlowJo software.

### Fluorescent Microscopy of mHsp70 Expression

LS174T and UD‐5 cells (5 × 10^4^ cells) were allowed to adhere to glass slides coated with poly‐L‐lysine (Sigma‐Aldrich) overnight. Cells were then incubated in a complete medium containing FITC‐conjugated cmHsp70.1  mAb for 30 min on ice, washed twice with PBS, and fixed with 4% w/v paraformaldehyde (PFA) in PBS for 15 min at RT. The cells were rewashed before being stained with phalloidin (1 µg mL^−1^) and DAPI (2 µg mL^−1^) for F‐actin and nuclei, respectively, for 1 h at RT in a light‐protected condition. Images were obtained using a fluorescence microscope with 63x oil objective (Leica, THUNDER Imager DMi8) equipped with Leica LAS X software.

### Cell Death Assay

Cell death was investigated using an Annexin V/PI apoptosis assay according to the manufacturer's protocol (ab14085, Abcam). Briefly, LS174T and UD‐5 target cells (2 × 10^5^ cells well^−1^) were co‐incubated with effector NK cells at a ratio of 1:1. After 8 h, cells were harvested and resuspended in a binding buffer containing Annexin V‐FITC and PI for 5 min at RT in the dark. Target cells were analyzed by flow cytometry after the exclusion of NK cells using APC‐conjugated anti‐CD45 (clone REA747; Miltenyi Biotec).

### Statistics

Statistical analysis was performed using GraphPad Prism software (version 9). The Kolmogorov‐Smirnov distribution test verified the distribution of the variables. The Student's t‐test or ANOVA compared normally distributed data, and non‐parametric distributions were compared using the Kruskal‐Wallis test. Results were presented as mean ± SD unless specified otherwise. The *p* values were considered statistically significant as ns: not significant, ^*^ *p* ≤ 0.05, ^**^ *p* ≤ 0.01, ^***^ *p* ≤ 0.001, and ^****^ *p* ≤ 0.0001.

## Conflict of Interest

Gabriele Multhoff is the founder and Chief Scientific Officer of multimmune GmbH. Alan Graham Pockley is Chief Executive Officer of multimmune GmbH; the authors declare no other conflict of interest.

## Author Contributions

G.M. and A.B.D. contributed equally to this work. M.Y.: conceptualization, methodology, writing–original draft. M.H.K, F.K.M, V.Z., R.O., M.K., S.H., C.C.H.: methodology. A.G.P., E.W., B.W.: review and editing. G.M.: conceptualization, supervision, funding acquisition, review, and editing. A.B.D.: conceptualization, methodology, supervision, review, and editing. All authors have read and agreed to the published version of the manuscript.

## Supporting information

Supporting Information

## Data Availability

The data that support the findings of this study are available from the corresponding author upon reasonable request.
