# Supplementary material for: Crosstalk Between NK Cell Receptors and Tumor Membrane Hsp70‐Derived Peptide: A Combined Computational and Experimental Study
Source: Adv Sci (Weinh). 2024 Jan 31;11(14):2305998. doi: 10.1002/advs.202305998 (PMC11005703; doi:10.1002/advs.202305998)
Supplement: Supplementary file 1 — Supporting Information [file ADVS-11-2305998-s001.pdf]

Supporting Information

for *Adv. Sci.*, DOI 10.1002/advs.202305998

Crosstalk Between NK Cell Receptors and Tumor Membrane Hsp70-Derived Peptide: A Combined Computational and Experimental Study

Mina Yazdi, Morteza Hasanzadeh Kafshgari, Fatemeh Khademi Moghadam, Vahid Zarezade, Rupert Oellinger, Mohammad Khosravi, Stefan Haas, Cosima C. Hoch, Alan Graham Pockley, Ernst Wagner, Barbara Wollenberg, Gabriele Multhoff and Ali Bashiri Dezfouli**

Crosstalk between NK Cell Receptors and Tumor Membrane Hsp70-Derived Peptide: A Combined Computational and Experimental Study

Mina Yazdi [1], Morteza Hasanzadeh Kafshgari [2], Fatemeh Khademi Moghadam [3], Vahid Zarezade [4], Rupert Oellinger [5,6], Mohammad Khosravi [7], Stefan Haas [8,9], Cosima C. Hoch [9],Alan Graham Pockley [10], Ernst Wagner [1], Barbara Wollenberg [9], Gabriele Multhoff [6,8,*,†], Ali Bashiri Dezfouli [6,8,9,*,†]

[1] Pharmaceutical Biotechnology, Department of Pharmacy, Ludwig-Maximilians-Universität (LMU), Munich, Germany.

[2] Heinz-Nixdorf-Chair of Biomedical Electronics, Campus Klinikum München rechts der Isar, TranslaTUM, Technische Universität München, Munich, Germany.

[3] Department of Biology, Faculty of Science, Shahid Chamran University of Ahvaz, Ahvaz, Iran.

[4] Behbahan Faculty of Medical Sciences, Behbahan, Iran.

[5] Institute of Molecular Oncology and Functional Genomics, School of Medicine, Technische Universität München, Munich, Germany.

[6] Central Institute for Translational Cancer Research (TranslaTUM), School of Medicine, Technische Universität München, Munich, Germany.

[7] Department of Pathobiology, Faculty of Veterinary Medicine, Shahid Chamran University of Ahvaz, Ahvaz, Iran.

[8] Department of Radiation Oncology, School of Medicine, Technische Universität München, Munich, Germany.

[9] Department of Otorhinolaryngology, School of Medicine, Technische Universität München, Munich, Germany.

[10] John van Geest Cancer Research Centre, School of Science and Technology, Nottingham Trent University, Nottingham, UK.

* Correspondence to: gabriele.multhoff@tum.de; ali.bashiri@tum.de

† These authors have contributed equally to this work

Supporting Information

PDB ID	Receptors	Docking Score (kcal/mol) (Schrödinger)	Docking Score (kcal/mol) (HPEPDOCK Server)
**2if7**	NTB-A (SLAMF6)	-9.845	-180.091
**3noi**	NKp30 (CD337)	-8.641	-172.299
**4s0u**	NKG2D (CD314)	-8.531	-158.982
**3bdw**	CD94/NKG2A/C	-8.045	-167.183
**1fm5**	CD69	-8.021	-167.62

Table S1. Molecular docking scores (kcal/mol) of TKD against different NK cell receptors. Molecular docking scores of the five top-ranked NK cell receptors (obtained by Schrödinger Software) interacting with TKD were calculated by the HPEPDOCK Server.

PDB ID	Receptors	Docking Score (kcal/mol) (HPEPDOCK Server)

**2if7**	NTB-A (SLAMF6)	-241.900
**3noi**	NKp30 (CD337)	-239.741
**4s0u**	NKG2D (CD314)	-212.440
**3bdw**	CD94/NKG2A/C	-235.560
**1fm5**	CD69	-235.522

Table S2. Molecular docking scores (kcal/mol) of Hsp70 against different NK cell receptors. Molecular docking scores of the five top-ranked NK cell receptors (based on the docking data of TKD-receptor) interacting with Hsp70 were calculated by the HPEPDOCK Server.

A)

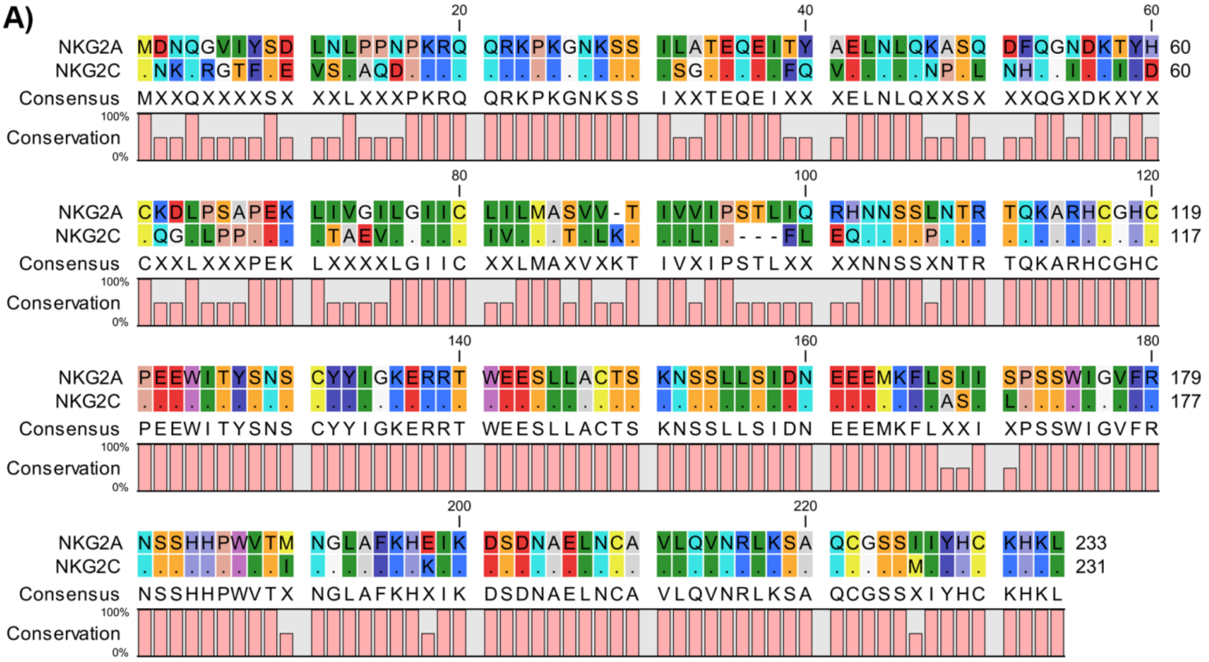

B)

NK-Cell Receptors	ACCESSION Number	Method	Max Score	Total Score	Query Cover	E value	Identities (%)	Positives	Gaps
**NKG2A**	AAL65234.1	Compositional matrix adjust.	364	364	100%	5e-134	180/234 (77%)	198/234 (84%)	4/234 (1%)
**NKG2C**	QSG30230.1								

Figure S1. Comparison of the protein sequences of NKG2A and NKG2C using the BLAST sequence analysis tool. A) NKG2A and NKG2C sequences aligned with the CLC Sequence viewer software. **B)** Sequence alignment summary table.

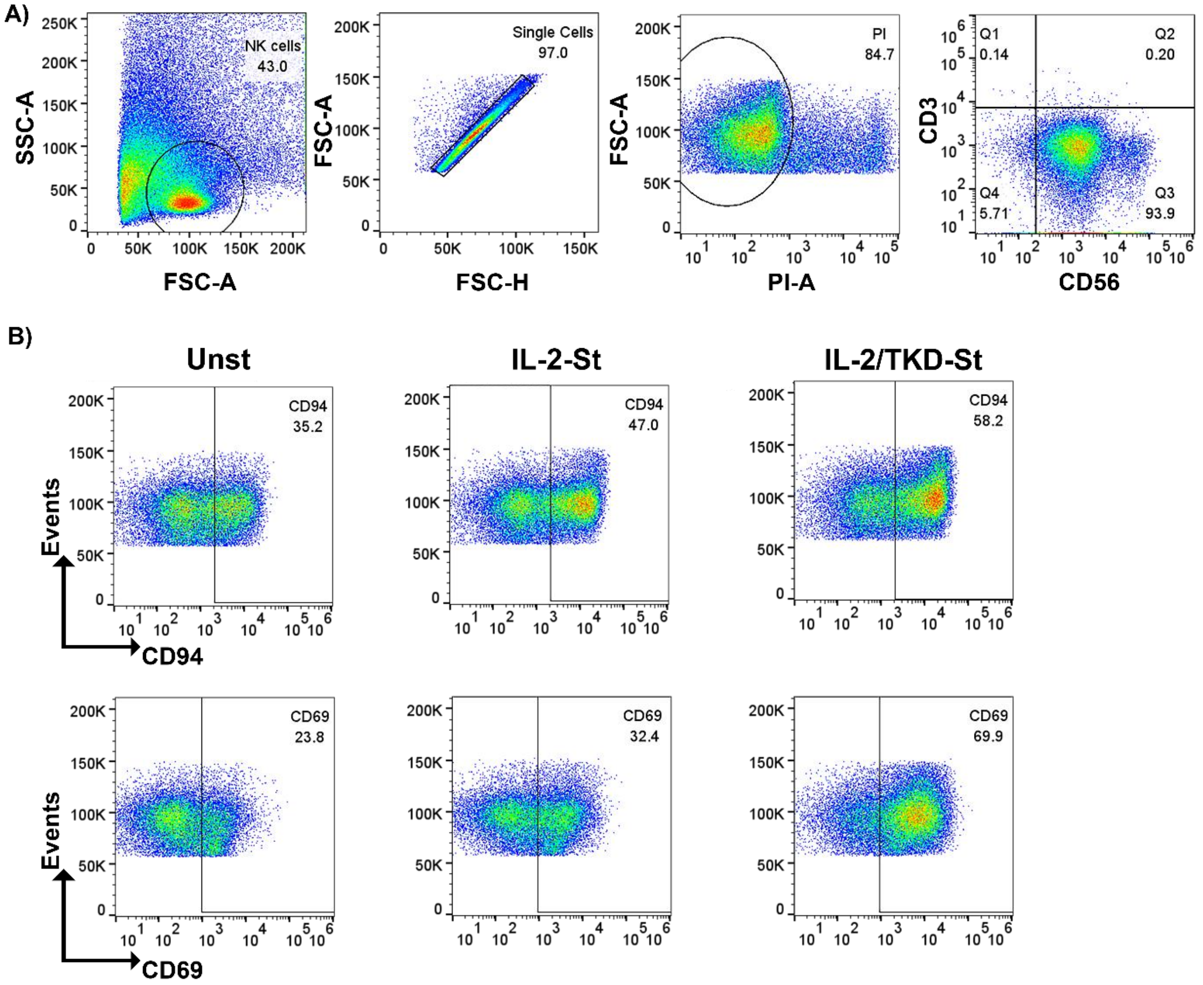

Figure S2. Representative example of a gating strategy to assess the CD69 and CD94 expression on unstimulated (Unst) and stimulated (St) $CD3^-/CD56^+$ NK cells by flow cytometry. PBMCs-derived NK cells were unstimulated or stimulated with IL-2 alone or in combination with TKD for 3 days at 37°C. The NK cell population was identified by side/forward scatter properties, single cell characteristics, viability (PI negative), and the cell surface markers $CD3^-/CD56^+/CD94^+$ or $CD3^-/CD56^+CD69^+$ using a panel of fluorescence-conjugated mAbs. The percentage of positively stained cells were determined respect to isotype-matched controls.

A)

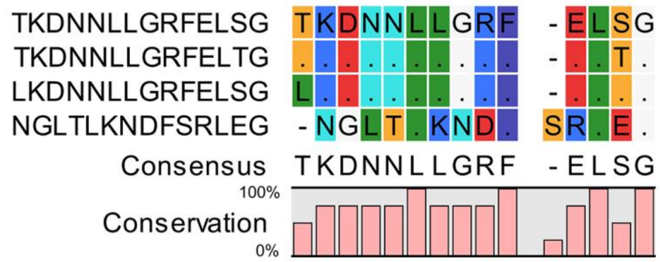

B)

Peptide	Docking score (kcal/mol)	3D	Ref.
TKDNNLLGRFELSG	-8.045	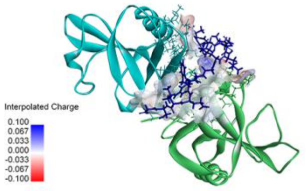	[1-3]
TKDNNLLGRFELTG	-7.016	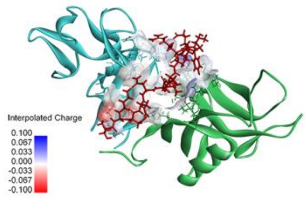	[1]
LKDNNLLGRFELSG	-6.121	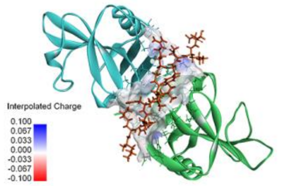	[2]
NGLTLKNDFSRLEG	-5.207	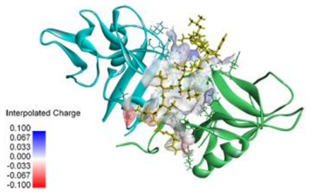	[3]

Figure S3. Comparison of the TKD with scrambled peptides in terms of their sequences and docking scores upon interaction with CD94/NKG2A/C. A) The scrambled sequences of TKDNNLLGRFELTG, LKDNNLLGRFELSG, and NGLTLKNDFSRLEG as well as TKD sequence aligned with the CLC Sequence viewer software. **B)** Molecular docking score (kcal/mol) of TKD and scrambled peptides against CD94/NKG2A/C protein receptor using Schrödinger Software. The schematic representations (3D) of binding interactions between the receptor active site and peptide residues were schematically represented (Cyan: CD94; Light green: NKG2A/C; Dark blue: TKD; Red: TKDNNLLGRFELTG; Orange: LKDNNLLGRFELSG; Yellow: NGLTLKNDFSRLEG).

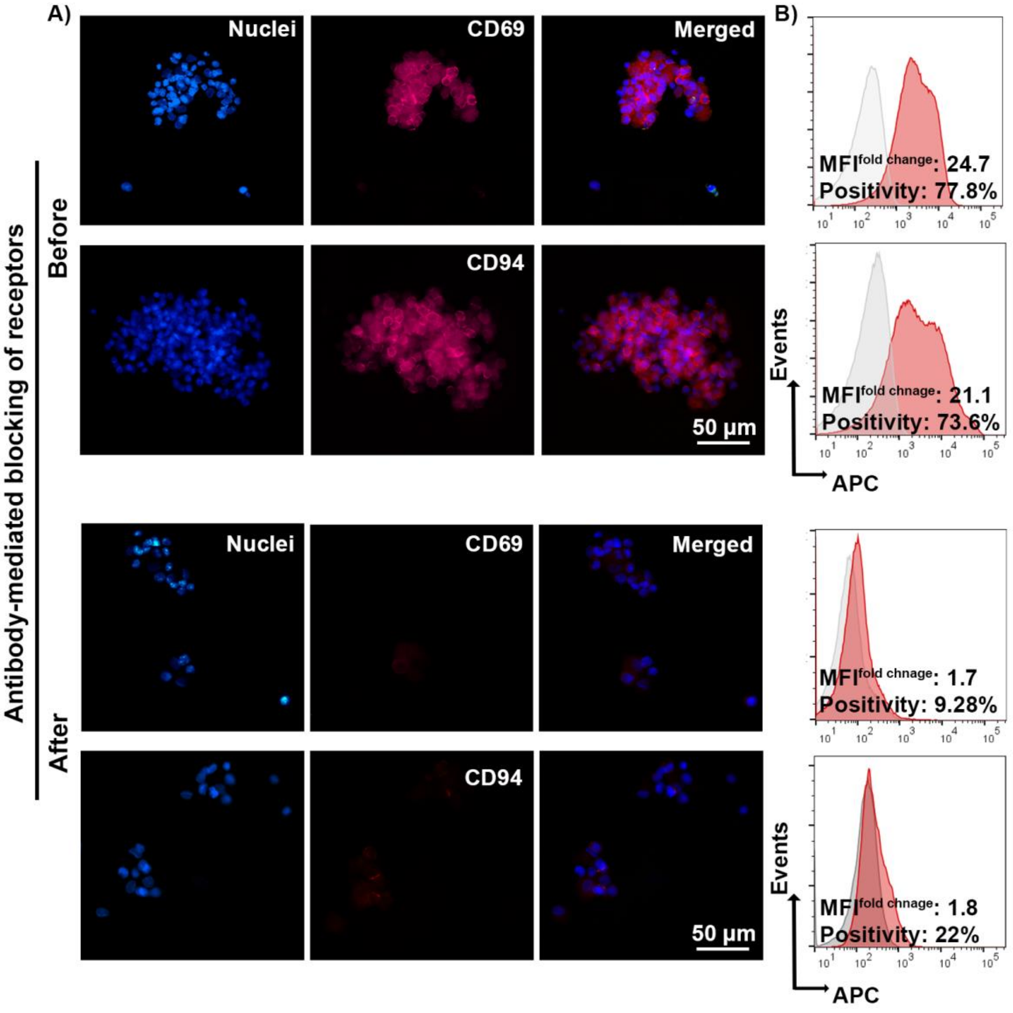

Figure S4. Expression of CD69 and CD94 receptors on the cell surface of IL-2/TKD-St NK cells before and after antibody blocking. NK cells were stimulated with IL-2/TKD for 3 days at 37°C at which time the expression of CD69 and CD94 was determined before and after antibody blocking. **A)** Cell surface expression of CD69 and CD94 visualized by fluorescence microscopy. DAPI (blue), CD69-APC, CD94-APC (red); scale bar, 50 µm. **B)** CD69 and CD94 expression determined by multiparameter flow cytometry (red histograms). Data are expressed as positively stained cells and MFI fold change compared to an isotype-matched control (gray histograms).

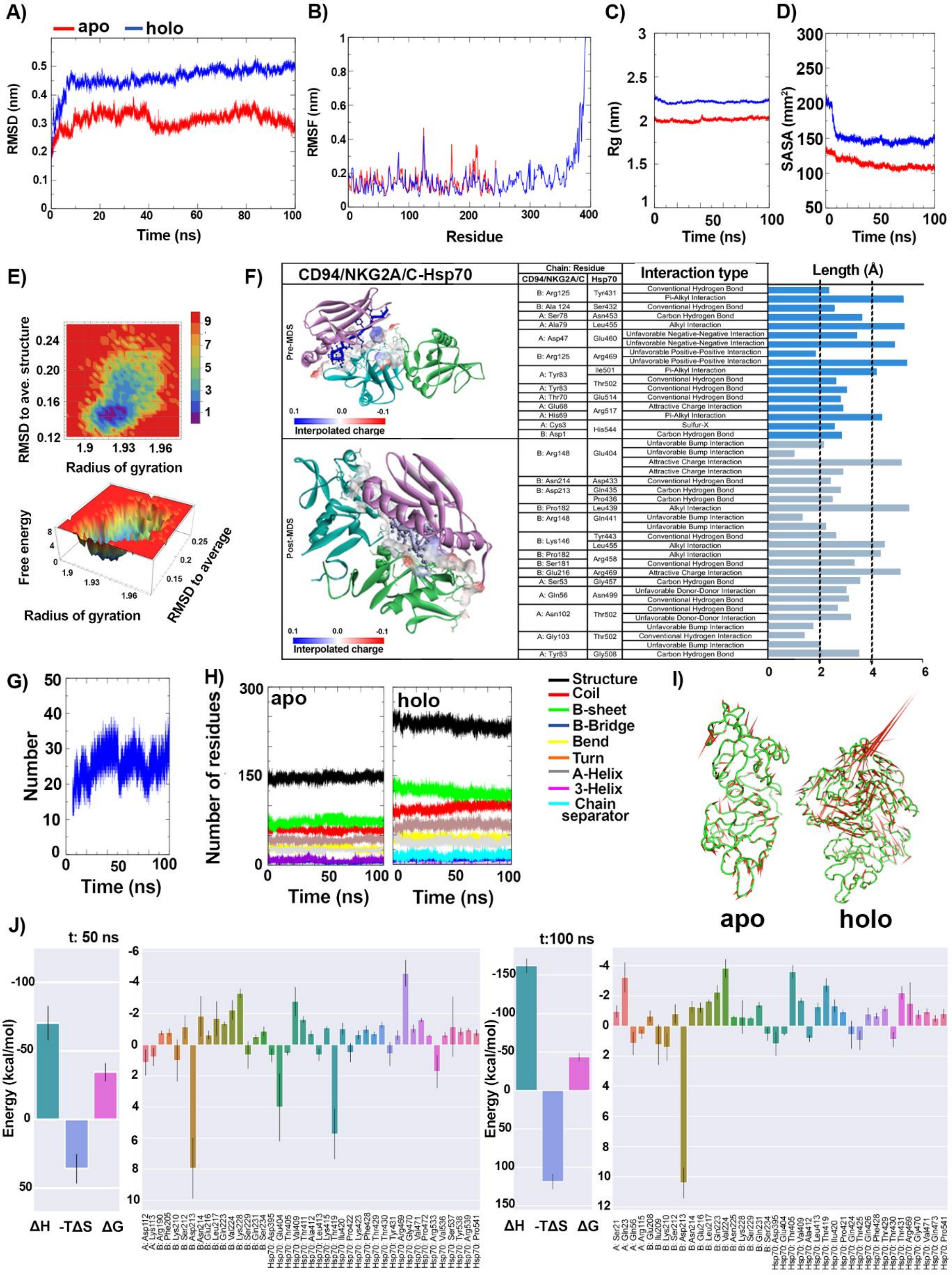

A)
apo
holo
RMSD (nm)
Time (ns)
B)
RMSF (nm)
Residue
C)
Rg (nm)
Time (ns)
D)
SASA (mm2)
Time (ns)
E)
RMSD to ave. structure
Radius of gyration
Free energy
RMSD to average
F)
CD94/NKG2A/C-Hsp70
Pre-MDS
Post-MDS
Interpolated charge
Chain: Residue
CD94/NKG2A/C
Hsp70
Interaction type
Length (Å)
G)
Number
Time (ns)
H)
apo
holo
Number of residues
Time (ns)
Structure
Coil
B-sheet
B-Bridge
Bend
Turn
A-Helix
3-Helix
Chain separator
I)
apo
holo
J)
t: 50 ns
t:100 ns
Energy (kcal/mol)
ΔH
-TΔS
ΔG

Figure S5. Molecular dynamics simulation. The complex of Hsp70 with CD94/NKG2A/C protein receptor was analyzed by MDS. For this, the apo (receptor without ligand) and holo (receptor-Hsp70 complex) structures were put into a 100-ns simulation time using GROMACS. **A)** RMSD values (nm), **B)** RMSF values (nm), **C)** Rg values (nm), and **D)** SASA values (nm) plotted against time (ns) for apo (red) and holo (blue) structures. **E)** The 2D and 3D FEL diagrams of holo receptor depicted as a function of Rg and RMSD. **F)** The schematic representations (3D) of binding interactions (including bond types and length (A°) between the receptor active site and Hsp70 residues in pre-MDS (Cyan: CD94; Light green: NKG2A/C; Light Purple: Hsp70 in which TKD sequence is presented with dark blue) and post-MDS (Cyan: CD94; Light green: NKG2A/C; Light Purple: Hsp70 in which TKD sequence is presented with light blue) complexes. The chains A and B refer to CD94 and NKG2A/C, respectively. **G)** H-bonds profile of holo form during MDS at 100 ns. **H)** DSSP presented as function of time for apo and holo structures. **I)** Porcupine plots of apo and holo structures obtained by PCA. **J)** The ΔG between the residues interacting in holo structure calculated by MM/GBSA using interaction entropy approximation of last 10 frames at 50 ns and 100 ns simulation. The letters A and B refer to CD94 and NKG2A/C, respectively.

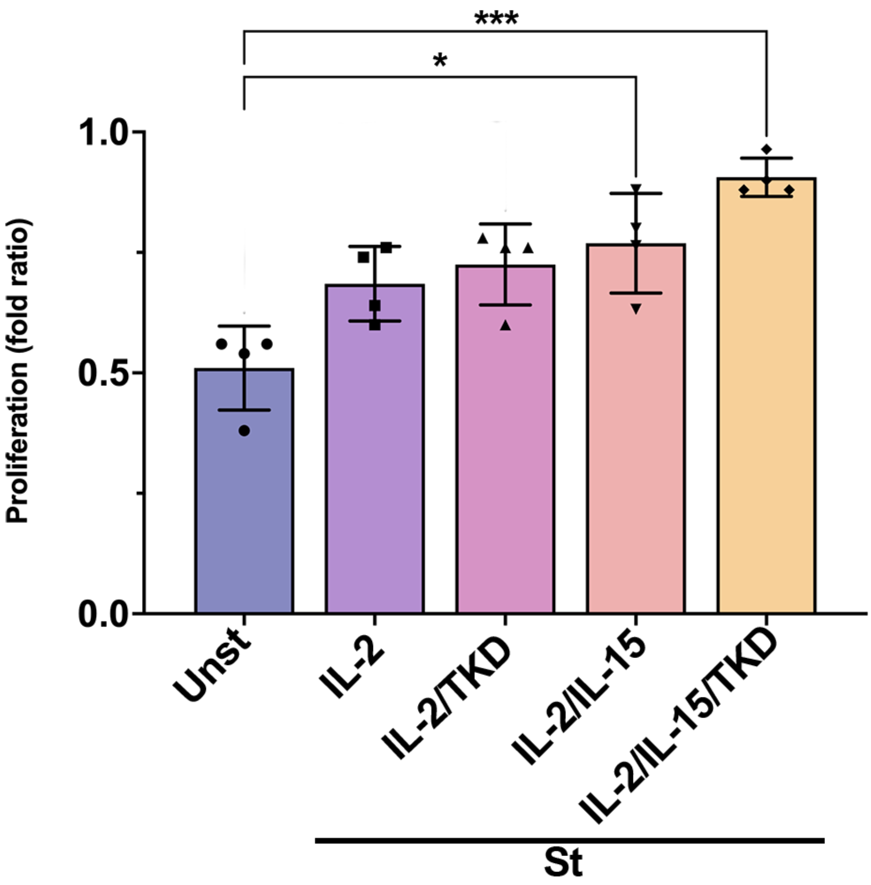

Figure S6. Expansion rates of NK cells after stimulation with IL-2, IL-2/TKD, IL-2/IL-15, and IL-2/IL-15/TKD. PBMC-derived NK cells seeded on day 0 at a cell density of 5 x 10^6 cells per well were

unstimulated or stimulated with IL-2, IL-2/TKD, IL-2/IL-15 and IL-2/IL-15/TKD. The numbers of viable cells were assessed on day 4 and compared to day 0. Data are mean fold change of triplicates of four different healthy donors ± SD (*p ≤ 0.05, ***p ≤ 0.001).

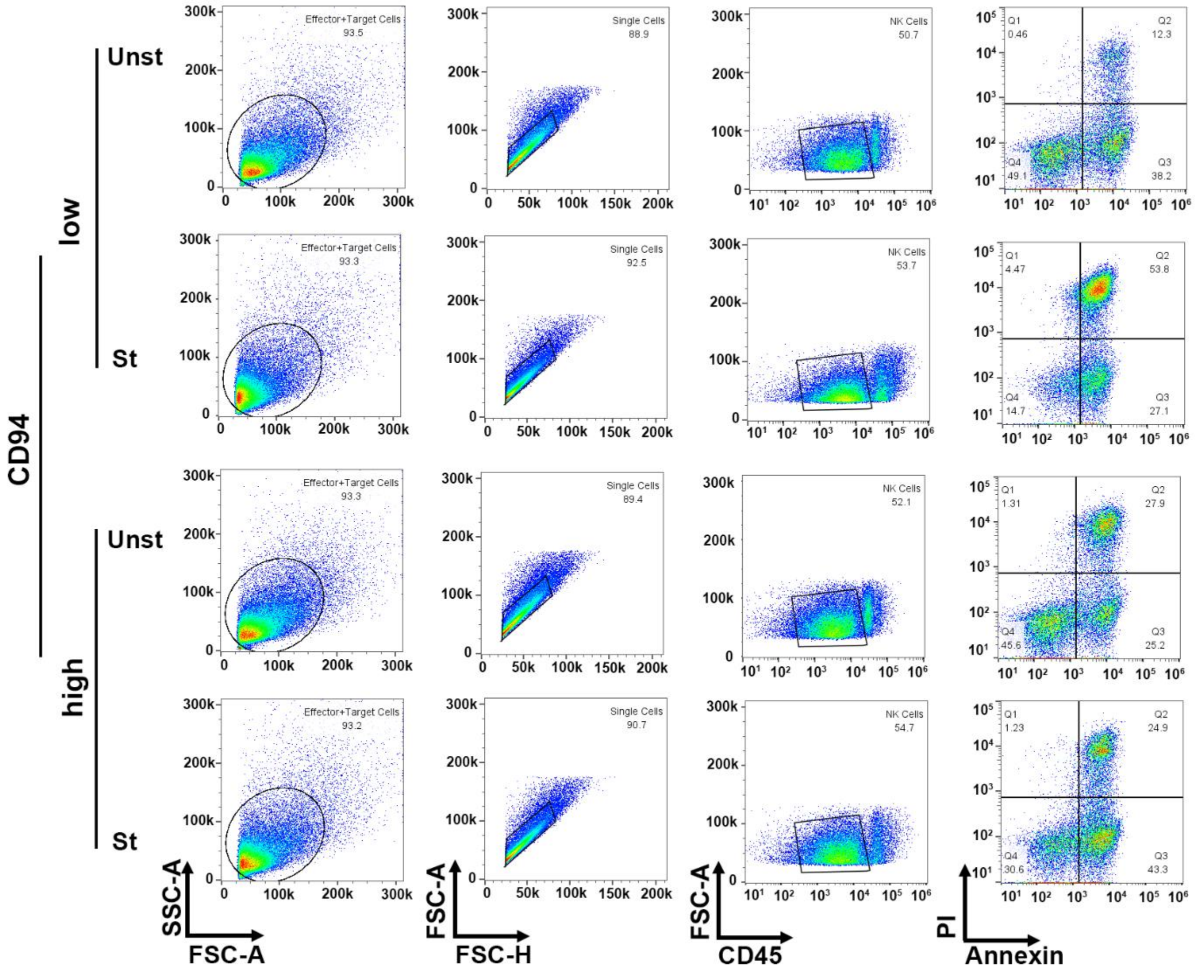

Figure S7. Representative example of gating strategy to evaluate apoptosis and/necrosis by an Annexin V/PI assay in colorectal cancer cells after an 8-hour co-incubation with IL-2/TKD-St NK cells. $CD94^{high}$ and $CD94^{low}$ NK cells that were unstimulated or stimulated with IL-2 or IL-2/TKD for 3 days at 37°C then co-cultured with LS174T cells for 8 hours. Effector and target cells were gated based on side/forward scatter properties, single cell characteristics, the leukocyte marker CD45, and the markers Annexin V-FITC vs PI. Early and late apoptosis in the target cell population was determined by a $CD45^{-}$/Annexin V^{+}/PI^{-} and a $CD45^{-}$/Annexin V^{+}/PI^{+} staining pattern, respectively.

References

[1] G. Multhoff, K. Pfister, M. Gehrmann, M. Hantschel, C. Gross, M. Hafner, W. Hiddemann, *Cell stress & chaperones* **2001**, *6* (4), 337.

[2] C. Gross, I. G. Schmidt-Wolf, S. Nagaraj, R. Gastpar, J. Ellwart, L. A. Kunz-Schughart, G. Multhoff, *Cell stress & chaperones* **2003**, *8* (4), 348.

[3] L. Elsner, P. F. Flügge, J. Lozano, V. Muppala, B. Eiz-Vesper, S. Y. Demiroglu, D. Malzahn, T. Herrmann, E. Brunner, H. Bickeböller, *Journal of cellular and molecular medicine* **2010**, *14* (4), 992.
